# Cutaneous T Cell Lymphoma: Targeting the Survival Network in Mycosis Fungoides and Sézary Syndrome

**DOI:** 10.3390/cancers18182956

**Published:** 2026-09-13

**Authors:** Philipp Heumann, Simon Mehler, Sara Martina Steinmann, Jan P. Nicolay, Martina Müller, Karsten Gülow

**Affiliations:** 1 Department of Internal Medicine I, Gastroenterology, Hepatology, Endocrinology, Rheumatology, Immunology, and Infectious Diseases, University Hospital Regensburg, 93053 Regensburg, Germany; philipp.heumann@ukr.de (P.H.); simon.mehler@stud.uni-regensburg.de (S.M.); sara.steinmann@ukr.de (S.M.S.); martina.mueller-schilling@ukr.de (M.M.); 2 Department of Dermatology, Venereology and Allergology, University Medical Center Mannheim, University of Heidelberg, 68167 Mannheim, Germany; jan.nicolay@umm.de

**Keywords:** cutaneous T cell lymphoma (CTCL), mycosis fungoides (MF), Sézary Syndrome, malignant T cell survival, NF-κB signaling, redox homeostasis, apoptosis resistance, targeted therapy, dimethyl fumarate (DMF), reactive oxygen species (ROS)

## Abstract

Cutaneous T cell lymphoma (CTCL) comprises a heterogeneous group of skin-homing T cell malignancies, with mycosis fungoides (MF) and Sézary Syndrome representing the most frequent and clinically relevant entities. Their clinical behavior differs substantially: MF often follows an indolent course over years, whereas Sézary Syndrome is a distinct clinicopathological entity that is typically characterized by erythroderma, a high burden of circulating malignant T cells and frequent lymph node involvement. Early-stage MF can often be controlled with skin-directed therapies, but advanced, relapsed or refractory CTCL remains therapeutically challenging. Established treatments are selected primarily according to disease stage, disease compartment and surface-target expression, whereas most pathway-directed, redox-modulating and apoptosis-sensitizing approaches remain investigational. Increasing evidence indicates that malignant T cells are maintained by an interconnected survival network rather than by a single dominant oncogenic pathway. This network includes constitutive inflammatory signaling, nuclear factor kappa B (NF-κB) activation, apoptosis resistance, altered redox homeostasis, mitochondrial and metabolic adaptation, rat sarcoma viral oncogene homolog (RAS)/rapidly accelerated fibrosarcoma (RAF)/mitogen-activated protein kinase kinase (MEK) signaling and epigenetic regulation. These mechanisms cooperate to promote malignant T cell survival, therapy resistance, and disease progression, but they also create opportunities for targeted therapeutic intervention. Particular emphasis is placed on redox-regulated cell death, nuclear factor kappa B (NF-κB)-dependent survival, B-cell lymphoma 2 (BCL-2) family proteins, RAS pathway alterations, epigenetic sensitization and mechanism-informed treatment strategies. Integrating clinicopathological staging with molecular profiling and functional vulnerability screens may support more rational combination therapies and improve patient stratification in CTCL.

## Simple Summary

Cutaneous T cell lymphoma (CTCL) is a rare form of blood cancer in which malignant T cells primarily involve the skin. Its clinical course varies widely: mycosis fungoides (MF), the most common form, often progresses slowly over many years, whereas Sézary Syndrome is usually more aggressive and can involve the skin, blood and lymph nodes. Advanced stages and relapsed disease remain difficult to treat. This is partly because malignant T cells use several mechanisms at the same time to survive, escape cell death and adapt to treatment pressure. These mechanisms include chronic survival signaling, changes in cell death pathways, disturbed control of oxidative stress, altered growth signaling and epigenetic changes. In this review, we discuss CTCL as a disease driven by such interconnected survival programs. Understanding this survival network may help researchers and clinicians to develop better treatment combinations and guide future efforts toward more personalized treatment strategies. Molecular profiling and functional testing are not yet used routinely for treatment selection, but may help identify suitable patient groups in specialized centers and clinical trials.

## 1. Introduction

Cutaneous T cell lymphoma (CTCL) comprises a heterogeneous group of non-Hodgkin lymphomas in which malignant T cells primarily involve the skin. The current classification framework is provided by the fifth edition of the World Health Organization Classification of Hematolymphoid Tumors (WHO-HAEM5) and the International Consensus Classification (ICC). Within this framework, primary CTCL are defined as lymphomas that present in the skin without evidence of extracutaneous disease at the time of diagnosis, and mycosis fungoides (MF) and Sézary Syndrome are recognized as distinct entities [1,2]. MF is the most common subtype and usually presents with patches, plaques or tumors, often after a long period during which the disease may resemble chronic inflammatory skin disorders. In many patients with early-stage MF, the clinical course is slow, and the disease can be controlled for long periods with skin-directed therapies. The Sézary Syndrome, in contrast, is a distinct clinicopathological entity with a typically aggressive clinical course, erythroderma, a leukemic burden of clonally related malignant T cells and frequent lymph node involvement [3,4,5]. It should be distinguished from erythrodermic MF with blood involvement, which may show overlapping clinical features but remains classified as MF. Accordingly, this review focuses primarily on mycosis fungoides and Sézary Syndrome, two distinct clinicopathological entities that represent the most frequent and clinically relevant CTCL entities and provide the strongest clinical and biological basis for the survival-network framework discussed here. Thus, CTCL does not describe a single clinical scenario, but a disease spectrum ranging from slowly progressive, skin-limited lymphoma to rapidly advancing systemic disease.

This heterogeneity is one of the main reasons why CTCL remains challenging in daily clinical practice. Early MF can be difficult to distinguish from benign inflammatory dermatoses, and diagnosis often requires careful clinicopathological correlation, immunophenotyping, T cell receptor (TCR) clonality testing and repeated biopsies over time. At the other end of the spectrum, advanced-stage MF and Sézary Syndrome are associated with impaired quality of life, limited long-term disease control and poor prognosis. In a large international cohort, advanced-stage MF and Sézary Syndrome were described as aggressive lymphomas with a median survival of 1 to 5 years, depending on clinical and biological risk factors [6]. Treatment decisions are therefore still strongly guided by disease stage, extent of skin involvement, tumor burden, blood involvement, lymph node status, and the general condition of the patient.

This stage-adapted approach is reflected in current international and national recommendations. The European Organisation for Research and Treatment of Cancer (EORTC) consensus update provides the most recent European treatment standard specifically for MF and Sézary Syndrome, whereas the European Society for Medical Oncology (ESMO), the National Comprehensive Cancer Network (NCCN) and German S2k recommendations provide broader clinical guidance for primary cutaneous lymphomas [4,7,8]. In addition, recommendations from the International Society for Cutaneous Lymphomas (ISCL), the United States Cutaneous Lymphoma Consortium (USCLC) and the EORTC have updated the framework for staging, response assessment and clinical trial design in primary cutaneous lymphomas [4]. In clinical practice, skin-directed therapies remain central in early-stage disease, whereas systemic therapies, antibody-based approaches, radiation, extracorporeal photopheresis and selected combination strategies are used in advanced, relapsed or refractory disease.

However, clinical stage alone does not fully explain treatment response or resistance. Many patients with advanced CTCL relapse after systemic therapy or achieve only transient disease control. This points to a central biological problem: malignant T cells can rely on several survival mechanisms at the same time. CTCL cells are usually not maintained by a single dominant oncogenic driver. Rather, they are shaped by interacting programs that include inflammatory signaling, resistance to programmed cell death, altered redox homeostasis, metabolic adaptation, oncogenic growth signaling and epigenetic regulation. Genomic and epigenomic studies support this view by demonstrating marked heterogeneity across CTCL subtypes and disease stages, together with recurrent alterations in pathways linked to T cell activation, apoptosis, nuclear factor kappa B (NF-κB) signaling, chromatin remodeling and DNA damage response [9,10,11].

**Figure 1:**
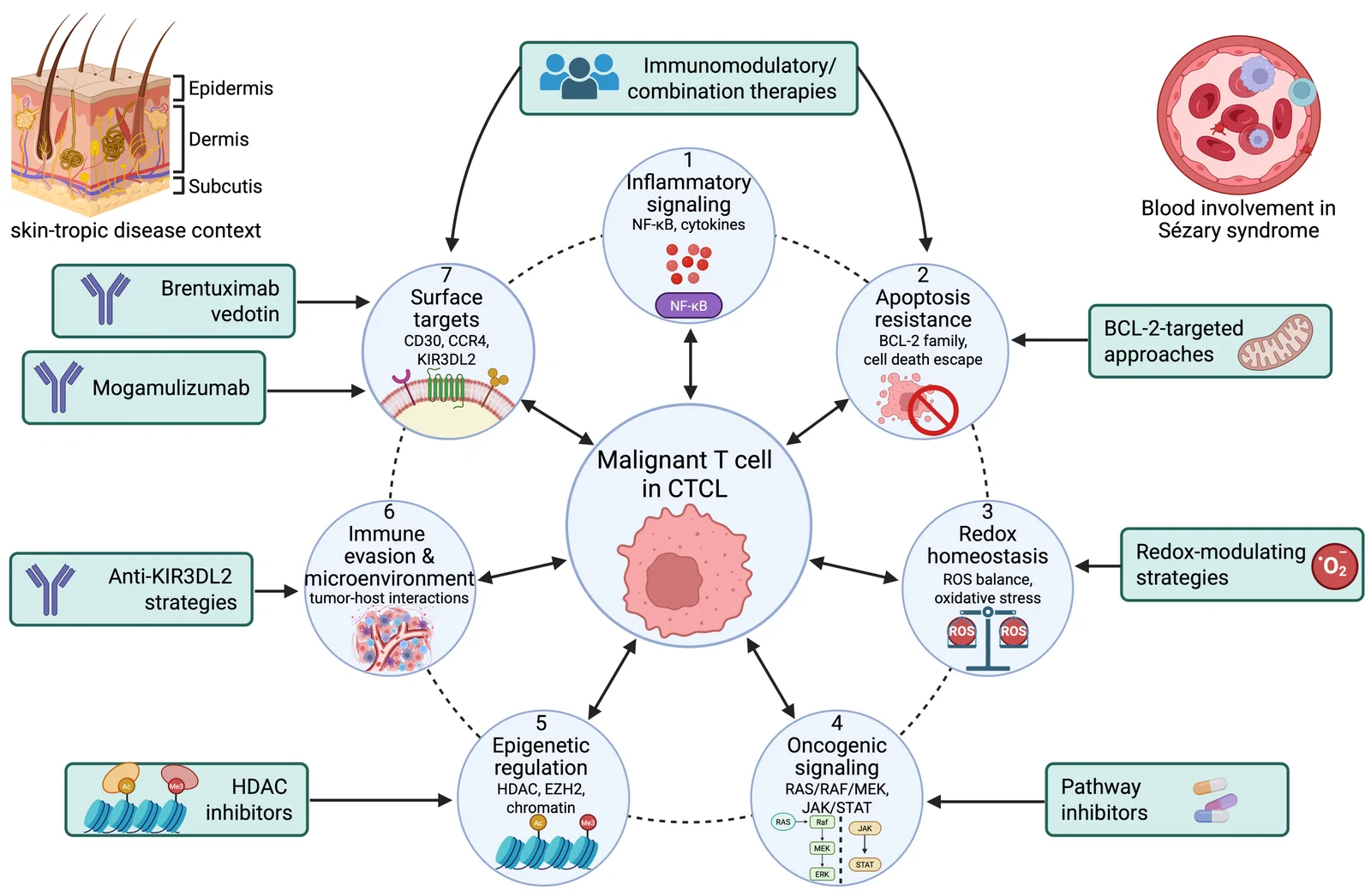
Targetable survival networks in cutaneous T cell lymphoma. Cutaneous T cell lymphoma (CTCL) is maintained by interconnected biological programs rather than by a single dominant oncogenic driver. Malignant T cells can rely on inflammatory signaling, nuclear factor kappa B (NF-κB) activation, apoptosis resistance, altered redox homeostasis, oncogenic signaling, epigenetic regulation, immune evasion, tumor–microenvironment interactions and surface antigen expression. These layers create therapeutic entry points. Some are addressed by established or guideline-supported treatments in appropriate clinical settings, including CD30- or CCR4-directed therapies and histone deacetylase (HDAC) inhibitors. By contrast, KIR3DL2-directed treatment and many pathway-directed, redox-modulating, B-cell lymphoma 2 (BCL-2)-targeted and combination approaches remain investigational or preclinical in CTCL. Created in BioRender. Pollinger, K. (2026) https://BioRender.com/yzgtgq8.

Together, these studies provide the rationale for viewing CTCL as a disease shaped by targetable survival networks rather than by a single dominant oncogenic event. This concept is supported by the molecular heterogeneity of CTCL and by recurrent alterations in pathways controlling T cell activation, NF-κB signaling, apoptosis, chromatin regulation and DNA damage response [9,10]. It is also reflected clinically by the development of targeted and immune-based therapies, including CD30-directed treatment with brentuximab vedotin, CC chemokine receptor 4 (CCR4)-directed treatment with mogamulizumab, histone deacetylase (HDAC) inhibition and emerging antibody approaches such as killer cell immunoglobulin-like receptor 3DL2 (KIR3DL2) targeting [12,13,14,15]. Nevertheless, many responses remain incomplete or temporary, underscoring the need for rational combinations and for a better understanding of the redundant pathways that support malignant T cell survival [16,17]. In this review, we summarize current concepts in the diagnosis and treatment of CTCL and discuss therapeutic opportunities across several biological layers, including surface antigen expression, immune evasion, inflammatory signaling, apoptosis resistance, redox homeostasis, oncogenic signaling, epigenetic regulation, and tumor–microenvironment interactions. By connecting clinical staging with molecular and functional vulnerability concepts, this review provides a framework for understanding how malignant T cell survival can be targeted in CTCL (Figure 1).

## 2. Diagnosis, Staging and Prognostic Factors: The Clinical Framework for Therapeutic Decisions

The diagnosis of CTCL requires an integrated assessment of clinical presentation, histopathology, immunophenotype and molecular findings. This is particularly important in early MF, where lesions may clinically and histologically mimic chronic inflammatory dermatoses. Patients often present with persistent or slowly progressive patches and plaques, frequently in sun-protected areas, and the diagnostic process may extend over several years. A single biopsy is often insufficient, and repeated sampling from clinically representative lesions may be required. Therefore, CTCL diagnosis should not rely on one isolated criterion, but on the careful correlation of clinical morphology, lesion distribution, histological features and ancillary tests [3,18]. The main diagnostic and staging domains that guide clinical decision-making in CTCL are summarized in Table 1.

Histologically, MF is typically characterized by epidermotropic atypical T cells, often with cerebriform nuclei, variable spongiosis and band-like or patchy lymphoid infiltrates in the upper dermis. However, these features may be subtle in early-stage disease. Immunohistochemistry supports the diagnosis by demonstrating a mature T cell phenotype, usually with expression of CD2, CD3 and CD5, and often a predominance of CD4-positive T cells. Loss of pan-T cell markers, particularly CD7 and sometimes CD26, can further support the diagnosis, although these findings are not entirely specific and must be interpreted in the clinical context. Demonstration of a clonal TCR rearrangement can provide additional evidence for CTCL, especially when identical clones are detected in separate biopsies or in skin and blood. Nevertheless, neither T cell receptor clonality nor loss of individual pan-T cell markers alone establishes malignancy, since restricted T cell populations and partial antigen loss may also occur in inflammatory dermatoses. The diagnosis of MF therefore requires concordant clinical, histopathological, immunophenotypic and molecular findings, often supported by repeated biopsies from representative lesions [4,19].

The Sézary Syndrome requires a distinct diagnostic approach since it is a separate clinicopathological CTCL entity. It is characterized by erythroderma and a substantial blood burden of clonally related malignant T cells, often accompanied by lymphadenopathy and severe pruritus. Although erythrodermic MF may also show blood involvement, it should not be considered synonymous with Sézary Syndrome; the distinction requires integration of the clinical course, skin histopathology, immunophenotype, flow-cytometric blood assessment and T cell receptor clonality testing [4,5]. Blood assessment is therefore essential in suspected Sézary Syndrome and in advanced CTCL. Flow cytometry is used to quantify aberrant circulating T cell populations, commonly CD4-positive cells with loss of CD7 and/or CD26. The demonstration of a dominant blood T cell clone, particularly when it is identical to the skin clone, provides additional support for the diagnosis.

Beyond conventional T cell receptor clonality testing, next-generation-sequencing (NGS)-based approaches are increasingly being applied in specialized centers and research settings to characterize CTCL. Targeted sequencing and broader genomic analyses have identified recurrent, but heterogeneous, alterations affecting T cell receptor and JAK/STAT signaling, NF-κB regulation, chromatin-modifying genes, tumor suppressor pathways and DNA damage response [9,10,20,21]. These findings currently complement rather than replace clinicopathological diagnosis, since no single genomic alteration is diagnostic for CTCL. Nevertheless, molecular profiling may support disease characterization, refine biological risk assessment and provide a bridge to the pathway-directed therapeutic concepts discussed in the following sections.

Within the ISCL/EORTC TNMB system, blood involvement is classified as B0, B1 or B2. Current EORTC recommendations favor flow-cytometric measurement of absolute counts of aberrant CD4^+^CD7^−^ or CD4^+^CD26^−^ T cells. B0 indicates no significant blood involvement, defined as <250 cells/µL; B1 indicates a low blood tumor burden of 250 to <1000 cells/µL; and B2 indicates a high blood tumor burden of ≥1000 cells/µL together with a demonstrable T cell clone. B0 and B1 may additionally be qualified according to the absence or presence of a blood clone. This standardized approach is more reproducible than manual Sézary cell counts and is relevant for staging, response assessment and treatment selection [5,22].

Staging provides the clinical framework for prognosis, treatment selection and response assessment. MF and Sézary Syndrome are staged according to the tumor–node–metastasis–blood (TNMB) system, which captures the extent of skin disease, lymph node involvement, visceral disease and blood tumor burden. The T category distinguishes patches or plaques involving <10% (T1) or ≥10% (T2) of the body surface area, one or more tumors (T3), and erythroderma involving ≥80% of the body surface area (T4). The N category is based on clinical and histopathological assessment of lymph nodes, whereas M0 and M1 indicate the absence or presence of visceral involvement, respectively. The B category reflects the blood classification described above and is particularly important in erythrodermic disease and Sézary Syndrome. This standardized staging approach allows clinicians to separate early-stage, skin-limited disease from advanced-stage disease with nodal, blood or visceral involvement, and it provides a common language for clinical trials [5].

Current guideline and consensus documents emphasize that treatment decisions in CTCL should be stage-adapted. For early-stage MF, skin-directed therapies such as topical corticosteroids, phototherapy, topical chlormethine, local radiotherapy and other local approaches are central. These treatments can provide long-term disease control in many patients and are usually preferred before systemic therapy is introduced. In contrast, advanced-stage MF and Sézary Syndrome often require systemic treatment strategies, including retinoids, interferon, extracorporeal photopheresis, histone deacetylase inhibitors, antibody-based therapies, radiation approaches and, in selected cases, chemotherapy or allogeneic stem-cell transplantation. Recent recommendations also incorporate targeted agents such as brentuximab vedotin for CD30-positive disease and mogamulizumab, particularly in patients with blood involvement or Sézary Syndrome [7,8,18].

Several guidelines provide complementary perspectives on diagnosis, staging, treatment and follow-up. The ESMO Clinical Practice Guidelines summarize diagnostic and therapeutic recommendations for primary cutaneous lymphomas in general [18]. The British Association of Dermatologists and U.K. Cutaneous Lymphoma Group guidelines provide detailed practical recommendations for multidisciplinary management [19]. The German S2k guideline offers an updated national consensus for cutaneous lymphomas [4], whereas the EORTC 2023 consensus update provides the most recent European treatment standard specifically for MF and Sézary Syndrome [7]. From a North American perspective, the NCCN Guidelines Insights summarize key updates for the diagnosis and management of primary cutaneous lymphomas [8]. Finally, the ISCL, USCLC and EORTC recommendations provide an updated framework for staging, response assessment and clinical trial design, which is essential for comparing outcomes across studies and for developing future therapeutic strategies [5]. In addition to diagnosis and staging, prognostic risk assessment has become increasingly relevant for therapeutic decision-making. The Cutaneous Lymphoma International Consortium identified clinical and biological prognostic markers in advanced-stage MF and Sézary Syndrome and developed a prognostic model for overall survival [6]. More recently, the Cutaneous Lymphoma International Prognostic Index (CLIPI) further refined risk stratification in advanced cutaneous lymphoma, supporting the integration of prognostic factors into treatment planning and patient selection for clinical studies [23].

Thus, diagnosis and staging remain the clinical backbone of CTCL management. They define disease category, risk, treatment intensity and follow-up strategy. At the same time, they do not fully capture the biological heterogeneity that drives treatment response and resistance. The next step is therefore to connect this clinical framework with the evolving therapeutic landscape and with molecular features that may help to refine patient stratification and treatment selection.

## 3. Current Treatment Landscape: From Skin-Directed Therapy to Systemic Targeted Approaches

Treatment of cutaneous CTCL is guided by disease subtype, stage, symptom burden, pace of progression, blood involvement, comorbidities, previous therapies and patient preference. In early-stage MF, the aim is usually not rapid systemic eradication, but durable disease control, relief of pruritus and preservation of quality of life. This differs from the treatment logic used for many aggressive nodal lymphomas. As early MF is often limited to the skin and may remain indolent for years, skin-directed treatment forms the backbone of management. Advanced-stage MF and Sézary Syndrome, in contrast, usually require systemic or multimodal strategies. Current recommendations therefore follow a stage-adapted approach, but also emphasize the need to balance response, durability, toxicity, treatment burden and quality of life [7,18]. The major established and emerging therapeutic approaches discussed in this section are summarized in Table 2.

In early-stage MF, topical and skin-directed therapies are usually the first therapeutic step. Topical corticosteroids are widely used because they can reduce inflammation, pruritus and lesion activity and are easy to integrate into long-term care. Phototherapy remains another central option. Narrow-band ultraviolet B (NB-UVB) is often used for patch-stage disease, whereas psoralen plus ultraviolet A (UVA) may be preferred for thicker plaques or more infiltrated lesions. Topical chlormethine has become an important treatment for limited or generalized skin involvement and is included in contemporary treatment recommendations. Its use is supported by randomized trial data and real-world studies showing clinical activity and improvement of skin disease burden in MF [24,38,39]. Local radiotherapy is highly effective for solitary, symptomatic or refractory lesions. Total skin electron beam therapy (TSEBT) is a specialized skin-directed option for patients with extensive, symptomatic or refractory cutaneous disease and can provide rapid reduction in skin tumor burden and pruritus. Conventional-dose TSEBT achieves high response rates but is associated with cumulative cutaneous toxicity and limited repeatability. Low-dose TSEBT, commonly delivered at 10–12 Gy, offers shorter treatment courses, lower toxicity and the possibility of retreatment; however, disease control is often not durable and TSEBT does not address blood, nodal or visceral involvement. It may therefore be used for cutaneous debulking or integrated with systemic therapy in selected patients [40]. In daily practice, these treatments are rarely chosen in isolation from practical considerations. Lesion morphology, extent of skin involvement, access to phototherapy, age, comorbidities and cumulative toxicity all influence the sequence and combination of skin-directed modalities [4,7,19].

The strength of skin-directed treatment is its ability to control disease with limited systemic toxicity. This is important because many patients with early MF need repeated, intermittent or maintenance therapy over many years. At the same time, skin-directed treatment has clear limitations. Responses may be partial, relapse after discontinuation is common, and some patients progress to plaques, tumors or erythroderma despite appropriate local therapy. Moreover, early-stage MF is not necessarily a minor disease from the patient’s perspective. Pruritus, visible lesions, sleep disturbance and the burden of chronic treatment can markedly affect quality of life. In CTCL cohorts, pruritus has repeatedly been identified as one of the most relevant patient-reported symptoms and is associated with impaired quality of life [41,42]. Early MF should therefore not be considered biologically or clinically trivial simply because it is often indolent. Treatment intensity has to be adjusted repeatedly to disease activity, symptoms and patient burden.

Systemic therapy is introduced when disease becomes extensive, symptomatic, tumor-stage, erythrodermic, blood-involved, relapsed or refractory to appropriate skin-directed treatment. Traditional systemic options include bexarotene, interferon-based therapy, low-dose methotrexate, extracorporeal photopheresis, histone deacetylase inhibitors and selected radiation-based approaches. Denileukin diftitox-cxdl (E7777), an interleukin-2 receptor-targeted fusion protein, has shown clinical activity in relapsed or refractory CTCL in a single-arm registrational study. Its availability and regulatory status vary between regions, and treatment requires monitoring for infusion-related reactions and capillary leak syndrome [43]. Bexarotene showed clinical activity in a multinational open-label phase II–III study of refractory advanced-stage CTCL, with a 45% response rate at the recommended starting dose of 300 mg/m^2^/day and improvements in pruritus and CTCL-specific quality of life [25]. Interferon-based therapy and bexarotene are often used as immunomodulatory approaches and may be combined with skin-directed modalities or extracorporeal photopheresis. Extracorporeal photopheresis has a particular role in erythrodermic CTCL and Sézary Syndrome, especially in patients with blood involvement, and is often used as part of combination treatment rather than as a rapidly cytoreductive monotherapy [26]. Low-dose methotrexate remains a pragmatic option in selected patients, although responses vary and monitoring is required.

Conventional chemotherapy has a limited role in CTCL because responses are often short-lived and cumulative toxicity can compromise subsequent treatment options. However, selected single-agent regimens, including gemcitabine and pegylated liposomal doxorubicin, may provide clinically meaningful disease control in relapsed or refractory CTCL and can be useful when more rapid cytoreduction is required. Pralatrexate has also shown activity in heavily pretreated CTCL, including transformed MF, but its use remains individualized and requires folate and vitamin B12 supplementation as well as careful monitoring for mucositis. Chemotherapy is therefore generally reserved for rapidly progressive tumor-stage disease, visceral involvement, transformation, bridging to allogeneic stem-cell transplantation, or highly refractory disease [16,44,45,46,47].

Targeted and immune-based therapies have become central elements of systemic treatment. Brentuximab vedotin, an antibody–drug conjugate directed against CD30, is the clearest example of a biomarker-linked therapy in CTCL. In the randomized phase 3 ALCANZA trial, brentuximab vedotin was superior to physician’s choice of methotrexate or bexarotene in previously treated CD30-positive CTCL [12]. Final analyses confirmed more durable responses and longer progression-free survival with brentuximab vedotin in CD30-positive cutaneous T cell lymphomas [27]. These data established CD30 expression as a clinically useful therapeutic marker. However, CD30 expression may vary between lesions and over time, and peripheral neuropathy can limit treatment duration. Careful tissue assessment and thoughtful sequencing are therefore essential.

Alemtuzumab represents a distinct therapeutic option from CD30- and CCR4-directed therapies. As a CD52-targeted antibody, it causes broad depletion of malignant and normal lymphocytes and is generally reserved for carefully selected patients with relapsed or refractory erythrodermic CTCL or Sézary Syndrome, particularly when blood involvement is prominent. Its use requires careful assessment of immune status and infection risk, as well as appropriate antimicrobial prophylaxis and monitoring for opportunistic infections and viral reactivation. Thus, potential clinical benefit must be weighed against the risk of profound and prolonged immunosuppression, relevant comorbidities and the overall treatment plan [4,7,28,29].

In contrast to surface-directed antibodies, immune checkpoint inhibition represents a distinct immunotherapeutic approach. In a multicenter phase II study, pembrolizumab showed clinical activity in relapsed or refractory MF and Sézary Syndrome, although immune-related toxicity and disease flares require careful monitoring. In malignant T cell diseases, however, programmed cell death protein 1 (PD-1)/programmed death-ligand 1 (PD-L1) signaling may affect both antitumor immunity and the malignant T cells themselves. PD-1 is expressed on malignant T cells in a subset of patients, and its functional consequences may be context-dependent; PD-1 blockade may enhance antitumor immune responses but could also influence malignant T cell activation or survival. This biological complexity may contribute to the transient disease flares observed in some patients, particularly those with Sézary Syndrome, and warrants cautious clinical interpretation. Pembrolizumab remains an investigational or off-label option in CTCL rather than an established standard treatment [30].

Mogamulizumab has changed treatment options particularly for patients with Sézary Syndrome or relevant blood involvement. The antibody targets CCR4, which is expressed by malignant T cells in many patients with MF and Sézary Syndrome. In the randomized phase 3 MAVORIC trial, mogamulizumab improved progression-free survival compared with vorinostat in previously treated MF and Sézary Syndrome [13]. Median investigator-assessed progression-free survival was 7.7 months with mogamulizumab compared with 3.1 months with vorinostat. The biological rationale is particularly strong in blood-involved disease, where circulating CCR4-positive malignant T cells are directly accessible. In clinical use, infusion reactions, rash and immune-mediated adverse events require attention. The timing of mogamulizumab before allogeneic transplantation also needs careful consideration because of its immunological effects.

Epigenetic therapy is another established systemic strategy. Vorinostat and romidepsin have both shown activity in relapsed or refractory CTCL and provided clinical proof of principle that chromatin regulation can be therapeutically exploited in malignant T cells [14,31]. Their effects are not limited to direct cytotoxicity; they can influence gene expression, apoptosis, immune regulation and cellular differentiation. Nevertheless, responses are heterogeneous, and fatigue, gastrointestinal symptoms, cytopenias, infections or cardiac concerns may restrict long-term use. These limitations have encouraged interest in more selective epigenetic approaches and in combinations that may increase sensitivity or delay adaptive resistance.

Dimethyl fumarate (DMF) is not guideline-supported or part of the standard treatment algorithm for CTCL and should therefore be considered an investigational, mechanism-informed therapeutic approach. Preclinical studies showed that DMF can interfere with NF-κB-dependent survival signaling, redox regulation and apoptosis resistance in CTCL models [33,35]. In a single-arm multicenter phase 2 study, oral DMF was evaluated for 24 weeks in 25 patients with relapsed or refractory CTCL. Among 23 evaluable patients, seven (30.4%) achieved a skin response, defined as a >50% reduction in the modified Severity-Weighted Assessment Tool score; blood responses were limited, and the study was not designed to establish comparative efficacy [34]. A subsequent translational study of DMF combined with extracorporeal photopheresis included four patients with Sézary Syndrome and reported clinical responses in skin and blood compartments, with time to next treatment ranging from 9 to 57 months across heterogeneous treatment courses [32]. However, the very small sample size, non-randomized design, concomitant and maintenance treatments, and heterogeneous prior therapy preclude conclusions regarding the efficacy or durability of this combination. DMF is therefore discussed in this review as an illustrative example of targeting inflammatory and redox-regulated survival pathways, rather than as an established CTCL treatment.

Several other investigational approaches aim to broaden the therapeutic repertoire. KIR3DL2 is of particular interest because it is frequently expressed in Sézary Syndrome and in subsets of MF. Lacutamab, an anti-KIR3DL2 antibody, showed clinical activity in early clinical evaluation and has supported further development of KIR3DL2-directed therapy [15]. Additional strategies include immune checkpoint modulation, pathway-directed inhibitors, apoptosis-targeting agents, redox-modulating strategies and rational combinations of biologic or targeted therapies. Importantly, not every relevant vulnerability in CTCL is defined by a recurrent mutation. Target expression, signaling state, microenvironmental interactions and stress-adaptation programs may be equally important for treatment response.

The growing number of treatment options also makes therapeutic sequencing more difficult. In early MF, escalation usually follows disease burden, symptoms and loss of control with local treatment. In advanced MF and Sézary Syndrome, treatment choice depends on blood involvement, CD30 expression, CCR4 expression, and pace of disease, prior therapy, infection risk, neuropathy, transplant eligibility and patient preference. Brentuximab vedotin is particularly relevant in CD30-positive disease, mogamulizumab is attractive in blood-involved disease, and extracorporeal photopheresis remains useful in erythrodermic disease and Sézary Syndrome, often in combination with immunomodulatory agents. Allogeneic stem-cell transplantation remains the only potentially curative option for selected fit patients with advanced disease. In contrast, chimeric antigen receptor (CAR) T cell therapy remains experimental in CTCL and should currently be considered only in early clinical development or clinical trial settings [48]. Long-term remissions have been reported in advanced CTCL, supporting a graft-versus-lymphoma effect, but treatment-related morbidity, timing and patient selection limit its use [36,37].

Overall, CTCL treatment has moved beyond a purely stage-based model. Clinical stage remains essential, but treatment increasingly also depends on disease compartment, target expression and biological vulnerability. Skin-directed therapies remain central in early MF, whereas advanced MF and Sézary Syndrome require systemic strategies that exploit surface antigens, immune regulation, epigenetic dependencies, inflammatory signaling and survival programs. Durable disease control remains difficult in advanced, relapsed or refractory CTCL. This unmet need provides the rationale for understanding CTCL not only as a clinically staged lymphoma, but also as a disease maintained by interconnected survival programs that may be targeted by rational therapeutic combinations.

## 4. Molecular Architecture of CTCL: Heterogeneity, Signaling Programs and Therapeutic Opportunities

### 4.1. Molecular Heterogeneity as a Biological Basis of Clinical Diversity

The clinical heterogeneity of cutaneous T cell lymphoma is mirrored by substantial molecular heterogeneity. MF and Sézary Syndrome share the defining feature of malignant skin-homing T cells, but they differ in clinical behavior, anatomical distribution, degree of blood involvement, immune dysfunction and therapeutic response. Early-stage MF often remains confined to the skin for long periods, whereas tumor-stage MF and Sézary Syndrome reflect biologically more aggressive disease states with increased tumor burden, systemic involvement or leukemic dissemination. These clinical differences are not explained by one universal genetic lesion. Unlike several other hematologic malignancies, CTCL is not defined by a single pathognomonic chromosomal translocation or a dominant oncogenic driver. Instead, genomic and functional studies have shown that malignant T cells can acquire survival advantages through several alternative and partially overlapping molecular routes. Importantly, MF and the Sézary Syndrome should not be regarded as sharing a uniform molecular architecture. MF is characterized by marked spatial and temporal heterogeneity across skin lesions and by molecular changes during progression, whereas the Sézary Syndrome more commonly displays a circulating malignant clone with systemic immune dysregulation and recurrent abnormalities in T cell activation, cell-cycle and chromatin-regulatory programs. These are tendencies rather than entity-defining genomic signatures, and substantial interpatient overlap remains [9,10,20,21].

This complexity is one of the major challenges in CTCL biology. Genomic studies have identified recurrent alterations in pathways involved in T cell activation, costimulatory signaling, cytokine signaling, NF-κB activation, apoptosis regulation, chromatin remodeling, DNA damage response and immune escape. Choi et al. performed exome, whole-genome and RNA sequencing of purified CTCL cells and identified mutations affecting genes linked to T cell activation, apoptosis, NF-κB signaling, chromatin remodeling and DNA damage response [9]. Integrated genomic analyses by Park et al. further supported the view that CTCL heterogeneity is shaped by diverse molecular drivers across MF, Sézary Syndrome and different disease stages [10]. Together, these studies provide a molecular explanation for a key clinical observation: patients with similar stage and histology may respond differently to the same therapy.

Several additional genomic studies have reinforced this concept. Whole-exome sequencing of Sézary Syndrome and other CTCL samples revealed recurrent alterations in genes involved in TCR signaling, NF-κB pathway regulation, chromatin modification and tumor suppressor control [20]. Genomic analysis of MF and Sézary Syndrome also identified recurrent alterations in tumor necrosis factor receptor 2 (TNFR2), encoded by TNFRSF1B, indicating that aberrant tumor necrosis factor receptor (TNFR) signaling may contribute to CTCL pathogenesis in subsets of patients [49]. In Sézary Syndrome, multiplatform genomic profiling further implicated dysregulation of cell-cycle checkpoints, T cell activation pathways and epigenetic regulators [21]. These studies differ in cohort composition and methodology, but they converge on a central conclusion that CTCL is molecularly heterogeneous and is maintained by interacting survival programs rather than by one uniform oncogenic event.

Beyond gene-level mutations, recurrent copy-number changes and structural abnormalities contribute to genomic instability and clonal evolution, particularly in advanced MF. Large-cell transformation is a clinically important manifestation of progression, but does not appear to result from a single uniform molecular event [9,10,20,21,50]. In parallel, immune-evasion mechanisms, including altered antigen presentation and suppression of antitumor immune responses, together with a Th2-polarized microenvironment, can reinforce malignant-cell survival. Recent single-cell and spatial analyses have begun to resolve the cellular and spatial organization of these interactions, including malignant T helper 2 (TH2)-like cells and B-cell-rich tumor niches; however, these findings remain primarily biologically informative and are not yet used routinely for treatment selection [51,52,53,54,55].

To provide a functional hierarchy for the survival-network concept, upstream lesions and contextual signals can be distinguished from downstream effector programs. Tumor-intrinsic alterations in T cell receptor/costimulatory signaling, JAK/STAT or TNF receptor pathways can converge on NF-κB, MAPK and related transcriptional programs. These downstream programs reinforce anti-apoptotic gene expression, metabolic and redox adaptation, and epigenetic plasticity, thereby raising the threshold for cell death. In parallel, cytokines, antigenic stimulation, and stromal- or immune-cell-derived signals from the cutaneous microenvironment can activate many of the same pathways without a corresponding tumor cell mutation. Thus, the network contains shared mechanisms across MF and Sézary Syndrome, including inflammatory signaling, apoptosis resistance and epigenetic plasticity, but their relative contribution differs by disease context. Early MF is often more dependent on the skin microenvironment, whereas Sézary Syndrome is characterized by systemic circulation of malignant cells, blood and nodal involvement, and more prominent immune dysregulation. With MF progression, tumor-intrinsic genomic and epigenetic alterations may increase cellular autonomy, although microenvironmental support remains relevant [9,10,50,51,52].

### 4.2. Recurrently Altered Pathways in Malignant T Cell Survival

Many of the recurrent alterations described in CTCL affect pathways that are normally required for T cell activation, proliferation and survival. This is important because malignant T cells do not need to acquire entirely new signaling programs; instead, they can use existing T cell pathways in a deregulated or persistent manner. Genomic studies have identified alterations in genes involved in T cell activation and proximal signaling, including phospholipase C gamma 1 (PLCG1), caspase recruitment domain family member 11 (CARD11), CD28 and related pathway components [9,10,20]. Functionally, PLCG1 mutations can increase downstream nuclear factor of activated T cells (NFAT) signaling, supporting the idea that altered proximal T cell signaling can directly affect transcriptional activation in CTCL [56]. Similarly, CD28 is a central costimulatory receptor controlling T cell proliferation, survival and effector function, and acquired CD28 mutations have been described in CTCL and other T cell neoplasms [57]. These alterations can feed into downstream NF-κB, NFAT, mitogen-activated protein kinase (MAPK) and cytokine-associated signaling pathways. Thus, CTCL biology remains closely linked to normal T cell activation biology, but with the critical difference that activation-associated survival signals become chronically maintained or insufficiently terminated.

Cytokine and Janus kinase (JAK)/signal transducer and activator of transcription (STAT) signaling form a second major layer of altered survival control. Activating alterations in STAT3, STAT5B, JAK1, JAK3 and cytokine receptor-associated pathways have been reported in CTCL and may contribute to proliferation, survival and immune dysregulation [9,20,21]. This pathway is particularly relevant because malignant T cells do not act in isolation, but remain embedded in a cytokine-rich skin and immune microenvironment. CTCL lesions are characterized by chronic inflammation, and cytokines produced by malignant T cells, reactive immune cells, keratinocytes and stromal components can contribute to local inflammation, impaired barrier function and tumor cell persistence [51,52]. In MF, this cytokine-rich tissue context may help to maintain local inflammation and support the persistence of malignant T cells within the skin. In Sézary Syndrome, malignant cells circulate between the blood, skin and lymph nodes, making cytokine responsiveness relevant not only for cutaneous inflammation, but also for systemic dissemination and immune dysregulation [52]. Importantly, the activation of JAK/STAT signaling does not necessarily require a mutation in the pathway itself. Malignant T cells can secrete cytokines that induce JAK1/STAT3-dependent effects in the skin microenvironment, and paracrine or autocrine cytokine stimulation may activate the same downstream transcriptional programs [58].

NF-κB signaling represents another central survival pathway in CTCL. Genetic alterations affecting proximal T cell signaling, costimulatory molecules, TNF receptor-associated pathways or CARD11-related signaling can converge on canonical or non-canonical NF-κB activation [9,20,49]. In addition, NF-κB can be activated by inflammatory cytokines, antigenic stimulation and microenvironmental signals, linking malignant T cell survival to the inflammatory context of CTCL lesions [51]. Functionally, constitutive NF-κB activity has been demonstrated in CTCL cell lines and patient-derived malignant T cells and has been associated with resistance to apoptosis [59,60]. Mechanistically, NF-κB can support expression of anti-apoptotic and pro-survival genes, thereby increasing the threshold for cell death. This concept is supported by studies showing that pharmacological or genetic interference with NF-κB signaling can restore apoptosis sensitivity in CTCL [33,35,60]. Moreover, combined inhibition of NF-κB and BCL-2 synergistically induced cell death in CTCL, directly linking inflammatory survival signaling to mitochondrial apoptosis control [17]. NF-κB therefore provides a direct mechanistic bridge between the genomic architecture of CTCL, its inflammatory microenvironment and therapeutic strategies that aim to lower the apoptotic threshold of malignant T cells.

Tumor suppressor and cell-cycle pathways are also recurrently affected, particularly in advanced or genomically unstable disease. Alterations involving TP53, the cyclin-dependent kinase inhibitor 2A (CDKN2A), ataxia telangiectasia mutated (ATM) and other DNA damage response or cell-cycle regulatory genes have been described in several CTCL cohort studies [9,10,20,21]. These alterations may facilitate genomic instability, clonal evolution and resistance to therapy-induced cell death. They are clinically relevant because they may contribute to progression from indolent skin-limited disease to tumor-stage or systemic disease. In early MF, malignant cells may still depend strongly on the inflammatory skin environment. During progression, additional genetic and epigenetic changes can support more autonomous growth, higher proliferative capacity and reduced apoptotic competence. Increasing intratumoral heterogeneity and branched clonal evolution have been described in MF and may contribute to disease progression and heterogeneous treatment responses [50].

Epigenetic deregulation adds a further layer of complexity. Genes involved in chromatin remodeling, DNA methylation and histone modification, including the DNA methyltransferase 3 alpha (DNMT3A), the AT-rich interaction domain 1A (ARID1A) and other epigenetic regulators, have been identified in genomic studies of CTCL [9,10,14,20,31]. Epigenetic changes can alter transcriptional states without requiring mutations in every downstream effector gene. This is particularly relevant in T cell malignancies, where activation state, lineage identity and differentiation programs are tightly controlled by chromatin structure. Epigenetic deregulation may therefore influence cytokine responsiveness, immune phenotype, apoptotic threshold and sensitivity to chromatin-directed therapies. This is clinically supported by the activity of histone deacetylase inhibitors in relapsed or refractory CTCL and biologically supported by studies identifying epigenetic regulators as modifiers of cell death sensitivity [14,31,61].

Taken together, the recurrently altered pathways in CTCL are not random. They repeatedly affect biological processes that normal T cells use for activation, survival, proliferation, cytokine response and adaptation to environmental cues. In malignant T cells, these programs become deregulated, persistent or insufficiently counterbalanced by cell death mechanisms. This explains why CTCL progression cannot be reduced to one pathway alone and why the same tumor cell may depend simultaneously on inflammatory signaling, cytokine responsiveness, apoptosis resistance, altered redox control and epigenetic plasticity.

### 4.3. Clonal Evolution, Disease Progression and Compartment-Specific Biology

The molecular architecture of CTCL should also be considered in the context of disease evolution. This is particularly relevant for MF, which often develops over many years and may pass through clinically distinct phases, ranging from patches and plaques to tumors and, in some patients, extracutaneous spread [62,63]. Early lesions usually contain a complex mixture of malignant T cells, reactive T cells, keratinocytes, dendritic cells, macrophages and stromal or inflammatory components. This cellular complexity contributes to the diagnostic difficulty of early MF and reflects the close interaction between malignant cells and the cutaneous microenvironment [3,53]. In this setting, the malignant clone may be difficult to distinguish from reactive T cell infiltrates and may remain dependent on tissue-specific inflammatory and survival signals. With progression, subclones with increased proliferative capacity, altered immune interactions, reduced apoptotic competence or greater independence from the local microenvironment may expand.

Genomic studies support such an evolutionary view of CTCL. Integrated genomic analyses have shown that molecular alterations differ between patients and disease stages, indicating that CTCL progression is not driven by one uniform molecular trajectory [10]. In MF, genomic analyses demonstrated extensive intratumoral heterogeneity and branched clonal evolution, with stage progression associated with increased intratumoral heterogeneity [50]. Single-cell analyses of advanced MF tumors further showed that the cutaneous tumor microenvironment is composed of malignant T cells together with diverse immune and stromal populations, including cell states associated with dysfunctional trafficking, altered antitumor immunity and matrix interactions [54]. This is clinically important because a single biopsy may not fully capture the molecular and cellular diversity of all lesions in one patient. Patches, plaques and tumors can coexist and may differ in cellular composition, proliferative activity, target expression and sensitivity to therapy.

The concept of clonal evolution also helps to explain why CTCL may become more difficult to treat over time. In early MF, malignant cells may still be strongly embedded in and supported by the inflammatory skin microenvironment. During progression, additional genetic, epigenetic and transcriptional changes may favor subclones with greater autonomy, increased resistance to cell death or altered interaction with immune cells [10,50]. This does not mean that advanced CTCL becomes independent of its environment. Rather, the relationship between malignant T cells and their surrounding tissue changes. The tumor clone may become more aggressive while still using cytokines, stromal signals and immune escape mechanisms to support persistence [51,52].

Compartment-specific biology is another important aspect. In skin-limited MF, the disease is largely shaped by the cutaneous microenvironment, which helps to explain why skin-directed therapies can be highly effective in early-stage disease [7,64]. In contrast, Sézary Syndrome is characterized by circulating malignant T cells, erythroderma and frequent lymph node involvement. In this setting, malignant cells interact not only with the skin but also with the blood and lymph node compartments [22,62]. These compartments may impose different selective pressures and may influence target expression, cytokine responsiveness and treatment sensitivity. Therefore, blood involvement is not only a staging parameter, but also a biological and therapeutic variable. This is supported by studies showing that blood tumor burden is relevant for staging, prognosis, response assessment and clinical management in MF and Sézary Syndrome [65,66].

This has direct therapeutic consequences. Therapies that are effective in skin-limited disease may be insufficient when malignant cells circulate in the blood or involve lymph nodes. Conversely, treatments that efficiently target circulating malignant cells may not fully eliminate skin-resident tumor cells or inflammatory niches. Mogamulizumab illustrates this principle, as CCR4-positive malignant T cells in the blood are directly accessible to antibody-based depletion, and clinical analyses have shown particularly relevant activity in blood-involved disease [13,67]. Extracorporeal photopheresis also fits into this compartment-oriented concept, because it is mainly used in erythrodermic CTCL and Sézary Syndrome, where blood involvement and immune modulation are central therapeutic considerations [26,68]. The dynamic and compartmentalized nature of CTCL has practical implications for molecular profiling. A skin biopsy taken at one time point may not represent all disease sites, and blood-based profiling may not fully reflect the biology of skin lesions. Re-biopsy, reassessment of blood involvement and repeated immunophenotypic or molecular evaluation may therefore become increasingly relevant in relapsed or progressive disease. This is especially important when therapeutic decisions depend on target expression, such as CD30, CCR4 or KIR3DL2, or when molecular vulnerabilities are considered for pathway-directed or combination therapies [15,22,65].

Taken together, CTCL should not be viewed as a static malignancy. It evolves over time, differs between disease compartments and may contain multiple subclones with distinct biological properties. This evolutionary and compartment-specific biology provides a rationale for repeated clinical and molecular reassessment and helps explain why durable disease control remains difficult in advanced MF and Sézary Syndrome. It also supports the broader concept that treatment selection should integrate clinical stage, disease compartment, target expression and functional vulnerability rather than relying on one parameter alone.

### 4.4. Targeting Survival Mechanisms in CTCL

Although genomic studies have provided important insight into CTCL biology, mutations alone do not fully explain therapeutic vulnerability. Several clinically relevant targets are defined by surface phenotype rather than by recurrent driver mutations. CD30 expression is a clear example. Brentuximab vedotin showed clinical activity in CD30-expressing CTCL in phase 2 studies and improved response durability and progression-free survival compared with physician’s choice in the randomized phase 3 ALCANZA trial [12,27,69]. Similarly, CCR4 can be exploited therapeutically by mogamulizumab, which improved progression-free survival compared with vorinostat in previously treated MF and Sézary Syndrome and showed particular relevance in blood-involved disease [13,70]. KIR3DL2 represents another surface-associated target, especially in Sézary Syndrome, and early clinical data with the anti-KIR3DL2 antibody lacutamab showed encouraging activity in relapsed or refractory CTCL [15]. These examples illustrate that a therapeutically relevant vulnerability may be defined by antigen expression, disease compartment and accessibility to antibody-based depletion rather than by a classical oncogenic mutation. Other therapeutic targets are intracellular and depend on signaling state, apoptotic priming, and redox control [71]. NF-κB activation is particularly important because it connects inflammatory signaling with apoptosis resistance and can influence the threshold for cell death [33,35,59,60,72]. Similarly, BCL-2 family proteins and epigenetic regulators can influence how malignant T cells respond to cell death-inducing therapies [17,32,61]. These mechanisms are discussed in more detail in the following sections.

RAS/RAF/MEK pathway dependence represents a more genotype-linked vulnerability. High-throughput mutation profiling identified KRAS and NRAS mutations in CTCL samples and showed that these alterations can sensitize tumor cells to inhibition of the RAS/RAF/MEK signaling cascade [73]. Subsequent work demonstrated that NRAS-mutated CTCL cells are sensitive to sorafenib-induced apoptosis, further supporting the idea that RAS pathway alterations may define a molecularly selected subgroup with pathway-dependent therapeutic sensitivity [73]. In contrast to surface-directed therapies such as brentuximab vedotin, mogamulizumab or lacutamab, RAS/RAF/MEK inhibition would rely on molecular selection of patients with pathway alterations.

Epigenetic regulation also contributes to functional vulnerability. Histone deacetylase inhibitors such as vorinostat and romidepsin have shown clinical activity in relapsed or refractory CTCL, demonstrating that chromatin-regulated transcriptional programs can be therapeutically targeted [31]. Beyond established histone deacetylase inhibition, epigenetic regulators may also influence cell death sensitivity. A clustered regularly interspaced short palindromic repeats-associated protein 9 (CRISPR-Cas9) screen identified enhancer of zeste homolog 2 (EZH2) inhibition as a sensitizer of malignant T cells to dimethyl-fumarate-induced cell death, linking epigenetic regulation to redox-dependent apoptosis sensitivity [61]. These findings indicate that epigenetic regulators can influence how CTCL cells respond to cell death-inducing therapies.

Overall, treatment response in CTCL is influenced by several factors that are not captured by stage and histology alone. These include surface antigen expression, blood involvement, intracellular signaling, apoptotic priming, redox state, genetic alterations and epigenetic regulation. This may explain why patients with similar clinical presentations can respond differently to the same therapy. It provides the biological framework for the treatment and combination concepts discussed in the following sections.

### 4.5. Clinically Relevant Biomarkers and Translational Stratification in CTCL

Clinically relevant biomarkers in CTCL include established surface antigens, measures of blood tumor burden, molecular features and emerging epigenetic markers. Their role differs according to the clinical context. Some biomarkers identify a directly targetable antigen, whereas others support diagnosis, staging, response assessment or biological disease characterization.

CD30 is the most established target-associated biomarker in CTCL. Its expression supports the use of the antibody–drug conjugate brentuximab vedotin, particularly in CD30-positive MF and primary cutaneous anaplastic large-cell lymphoma [12,27]. CCR4 expression provides the biological basis for treatment with mogamulizumab and is particularly relevant in blood-involved MF and Sézary Syndrome [13,70]. KIR3DL2 represents another promising surface marker, especially in Sézary Syndrome, and may identify patients for treatment with the anti-KIR3DL2 antibody lacutamab [15]. However, surface-target expression may vary between patients, lesions and disease compartments and should therefore be reassessed when clinically relevant.

Blood tumor burden is both a staging variable and a clinically meaningful biomarker. Flow-cytometric quantification of aberrant circulating T cell populations, supported by T cell receptor clonality assessment where appropriate, contributes to diagnosis, classification and response monitoring, particularly in Sézary Syndrome [7,8,22]. Blood involvement also has therapeutic implications, since mogamulizumab has shown particular clinical activity in patients with circulating malignant T cells [13,70]. Repeated assessment of blood disease can therefore complement skin-based response evaluation and support treatment decisions in multi-compartment disease.

Molecular profiling has substantially improved the understanding of CTCL heterogeneity, although its routine value for selecting systemic therapy remains limited. Recurrent alterations in pathways related to T cell activation, JAK/STAT and NF-κB signaling, apoptosis control, DNA damage response and chromatin regulation may define biologically distinct disease subsets [9,10,20,21]. At present, these alterations are primarily informative for disease characterization and for the development of biomarker-guided clinical trials. Their clinical utility may increase when molecular data are integrated with disease compartment, surface-target expression and functional drug-sensitivity testing.

Emerging epigenetic biomarkers may add a further translational layer. Aberrant DNA methylation patterns and chromatin-regulatory alterations have been associated with CTCL and Sézary Syndrome biology [11,74]. In addition, CTCL cells express human telomerase reverse transcriptase (hTERT) and show disease-associated patterns of TERT promoter methylation [75]. These markers are not yet established for routine treatment selection. However, they may help define epigenetically distinct disease states, monitor malignant-cell persistence or identify patients more likely to benefit from chromatin-directed therapies. Future biomarker strategies in CTCL will therefore likely require integration of surface-target expression, blood tumor burden, molecular alterations and functional vulnerabilities rather than reliance on a single marker.

For pathway-directed approaches, several measurable parameters may provide a basis for translational stratification, although none are currently validated for routine treatment selection. NF-κB pathway activity can be assessed in tumor samples by nuclear p65/RELA localization, phospho-protein assays or pathway-related gene-expression signatures. Redox dependence may be explored through markers of thioredoxin or glutathione metabolism and oxidative-stress response programs, whereas apoptotic priming can be assessed by BCL-2-family protein expression and functional BCL-2 homology 3 (BH3) profiling. Epigenetic vulnerability may be captured by DNA methylation profiling, chromatin-regulatory alterations or histone-modification patterns. At present, these approaches are best suited to specialized centers and biomarker-guided clinical trials, where they may help enrich molecularly or functionally defined patient subsets for pathway-directed combinations rather than determine standard treatment outside a trial setting [10,22,32,35,61,65].

## 5. Inflammatory Signaling, NF-κB and Apoptosis Resistance in Malignant T Cells

### 5.1. Inflammation as a Disease-Promoting Context in CTCL

Inflammation is a prominent feature of CTCL lesions and is not only a secondary reaction to malignant infiltration. In MF, malignant T cells reside in chronically inflamed skin, where they interact with keratinocytes, dendritic cells, macrophages, fibroblasts and reactive lymphocytes. In Sézary Syndrome, malignant T cells circulate between blood, skin and lymphoid tissues and are exposed to both systemic and tissue-specific inflammatory signals. This inflammatory context is clinically visible as erythema, pruritus, barrier dysfunction and recurrent infections, but it also has biological relevance because cytokines and cell–cell interactions can support tumor cell persistence [51,52].

The inflammatory environment of CTCL is formed by both malignant and non-malignant cells. Malignant T cells can produce cytokines and chemokines that recruit and modify surrounding immune cells, whereas keratinocytes and stromal cells respond by producing additional mediators that maintain local inflammation. This creates a feed-forward situation in which malignant cells and the tissue environment reinforce each other. Such mechanisms are relevant in early MF, where the malignant clone may still be strongly dependent on the cutaneous microenvironment, but they also remain important in advanced disease, where tumor cells acquire additional genetic and epigenetic alterations while continuing to use inflammatory signals for survival [51,58].

Several studies have linked cytokine signaling directly to CTCL pathogenesis. STAT3 was shown to regulate spontaneous interleukin (IL)-5 production and IL-13 expression in CTCL cell lines, suggesting that aberrant STAT signaling can contribute to the cytokine phenotype of malignant T cells [76]. Malignant CTCL cells can also express IL-17 through a JAK3/STAT3-dependent mechanism, providing a direct connection between oncogenic signaling and inflammatory cytokine production [77]. More recently, malignant T cells were shown to induce skin barrier defects through cytokine-driven JAK1/STAT3 signaling in the skin microenvironment, supporting the concept that malignant T cell signaling can directly affect tissue function [58]. These findings help explain why inflammation, barrier dysfunction and tumor cell survival are closely linked in CTCL.

### 5.2. NF-κB as a Link Between Inflammation and Survival

NF-κB is one of the best-characterized molecular links between inflammation and apoptosis resistance in CTCL. Early studies showed constitutive NF-κB activity in CTCL cells and MF lesions, suggesting that this pathway is active in malignant T cells rather than merely reflecting reactive inflammation [59,78,79]. Subsequent work further supported the role of NF-κB in CTCL cell survival and apoptosis resistance. Sors et al. showed that constitutive activation of the canonical NF-κB pathway is linked to the expression of anti-apoptotic genes and that proteasome inhibition can interfere with this survival axis [60]. Together, these studies established NF-κB as a functionally relevant pathway in CTCL rather than only as a marker of inflammation.

NF-κB can be activated through several routes in CTCL. Genetic alterations affecting proximal T cell receptor signaling, costimulatory molecules, CARD11-related signaling or TNF receptor-associated pathways can converge on NF-κB activation. In addition, cytokines, microbial products, antigenic stimulation and stromal signals can activate NF-κB without requiring a mutation in the pathway itself [9,20,49]. Thus, NF-κB activation may arise from both tumor-intrinsic alterations and extrinsic inflammatory cues, which are difficult to separate within CTCL lesions but may jointly contribute to malignant T cell persistence. The biological consequences of NF-κB activation fit well with the clinical behavior of CTCL. NF-κB regulates genes involved in inflammation, cytokine production, cell survival and resistance to apoptosis. In malignant T cells, this can increase the threshold for death receptor-mediated or mitochondria-dependent apoptosis. NF-κB function in CTCL has therefore been discussed as a central mechanism linking inflammatory signaling to therapy resistance [72]. This concept is also supported by experimental studies showing that the downregulation of NF-κB signaling can sensitize CTCL cells to apoptosis. Curcumin, for example, induced apoptosis in CTCL cells in association with the inhibition of STAT3 and NF-κB signaling [80].

### 5.3. Apoptosis Resistance in CTCL

Resistance to apoptosis is a recurring feature of CTCL and is relevant for both disease persistence and treatment failure. In normal T cell biology, programmed cell death is essential to terminate immune responses and prevent the uncontrolled expansion of activated lymphocytes [81,82]. Activated T cells are normally eliminated through tightly regulated mechanisms such as activation-induced cell death, death receptor-mediated apoptosis and mitochondrial apoptosis [81,83,84]. Malignant T cells can acquire defects in these pathways and thereby escape physiological control mechanisms that would normally limit T cell survival [85,86,87].

Several studies have shown that CTCL cells are resistant to activation-induced cell death and CD95-mediated apoptosis. Ni et al. reported that resistance to activation-induced cell death and bystander cytotoxicity through the CD95/CD95 ligand (L) pathway is involved in CTCL pathogenesis [87]. Klemke et al. further showed that impaired T cell receptor-induced signaling is crucial for defective CD95L upregulation and protects CTCL cells from activation-induced cell death [86]. This is particularly relevant because it links altered T cell signaling, which is already a recurring theme in CTCL genomics, to a concrete defect in apoptotic control. Defects in the extrinsic apoptosis pathway have also been described downstream of death receptor engagement. Braun et al. showed that death receptor-mediated apoptosis can be blocked early in the extrinsic cascade in CTCL cells, at the level of death-inducing signaling complex formation [88]. Together with the findings by Ni et al. and Klemke et al., this supports the view that malignant T cells are protected at several levels of the extrinsic apoptosis pathway: insufficient induction of death receptor ligands, impaired activation-induced cell death and defective signal transmission after death receptor engagement [86,87,88].

Apoptosis resistance is not limited to death receptor pathways. Mitochondrial apoptosis control is also relevant, particularly through anti-apoptotic BCL-2 family members. Increased anti-apoptotic buffering can protect malignant T cells from cell death induced by cellular stress, therapy or immune attack. This provides a rationale for combining treatments that reduce inflammatory survival signaling with agents that lower mitochondrial apoptosis resistance. Combined inhibition of BCL-2 and NF-κB synergistically induced CTCL cell death and reduced tumor growth in preclinical models [17]. These findings suggest that CTCL cells can become more sensitive to cell death when NF-κB-driven survival signaling and mitochondrial apoptosis control are targeted together.

Taken together, CTCL cells can evade apoptosis at several points. They may fail to induce CD95L after T cell receptor stimulation, resist death receptor-mediated apoptosis and increase mitochondrial anti-apoptotic buffering. These defects help explain why malignant T cells can persist in chronically inflamed skin and blood despite immune activation and therapeutic pressure. They also provide a rationale for treatment strategies that do not only inhibit proliferation, but actively restore apoptosis sensitivity.

### 5.4. Targeting NF-κB-Driven Survival Signaling

The functional importance of NF-κB in CTCL makes this pathway therapeutically attractive, but direct NF-κB inhibition is challenging because NF-κB also has essential physiological roles in immune regulation, inflammation and tissue homeostasis. Therapeutic strategies therefore often aim to interfere with upstream activators, downstream survival factors or regulatory mechanisms that keep NF-κB-dependent survival programs active in malignant T cells. Proteasome inhibition is one example. Sors et al. showed that CTCL cells display constitutive activation of the canonical NF-κB pathway and that proteasome inhibition can reduce NF-κB activity, downregulate anti-apoptotic genes and induce apoptosis [60]. Similarly, inhibition of STAT3 and NF-κB signaling by curcumin induced apoptosis in CTCL cell lines and patient-derived cells, supporting the concept that inflammatory survival signaling can be therapeutically targeted in malignant T cells [80]. DMF is particularly relevant in this context because it links anti-inflammatory activity, redox biology and apoptosis sensitization. In CTCL models, DMF inhibited NF-κB signaling, restored apoptosis sensitivity and reduced tumor growth and metastasis [33]. The work by Schroeder et al. provides an additional mechanistic layer to this concept. In CTCL cell lines, NF-κB activity was linked to increased expression of the inhibitor of apoptosis proteins (IAP) cIAP1, cIAP2 and XIAP. These proteins can interfere with the formation of the Ripoptosome and thereby prevent the induction of apoptosis and necroptosis. Targeting thioredoxin-1 by DMF reduced this NF-κB-dependent survival program and induced Ripoptosome-mediated cell death [35]. This is important since it shows that NF-κB does not only regulate general inflammatory gene expression in CTCL cells, but can directly maintain a molecular block against regulated cell death. This mechanism also connects NF-κB signaling with redox control [71]. DMF can modify redox-sensitive proteins, and thioredoxin-1 is a central regulator of cellular redox homeostasis. By targeting thioredoxin-1, DMF affects a redox-regulated survival axis that intersects with NF-κB activity and IAP expression [35,89]. This provides a plausible explanation for why CTCL cells, which are protected by NF-κB-dependent anti-apoptotic programs, can become vulnerable when redox control and inflammatory survival signaling are disturbed at the same time.

The concept of targeting NF-κB-driven survival signaling also supports rational combinations. One example is the combination of NF-κB inhibition with BCL-2 targeting. Froehlich et al. showed that combined inhibition of BCL-2 and NF-κB synergistically induces cell death in CTCL, indicating that inflammatory survival signaling and mitochondrial apoptosis control cooperate in maintaining malignant T cell survival [17].

**Figure 2:**
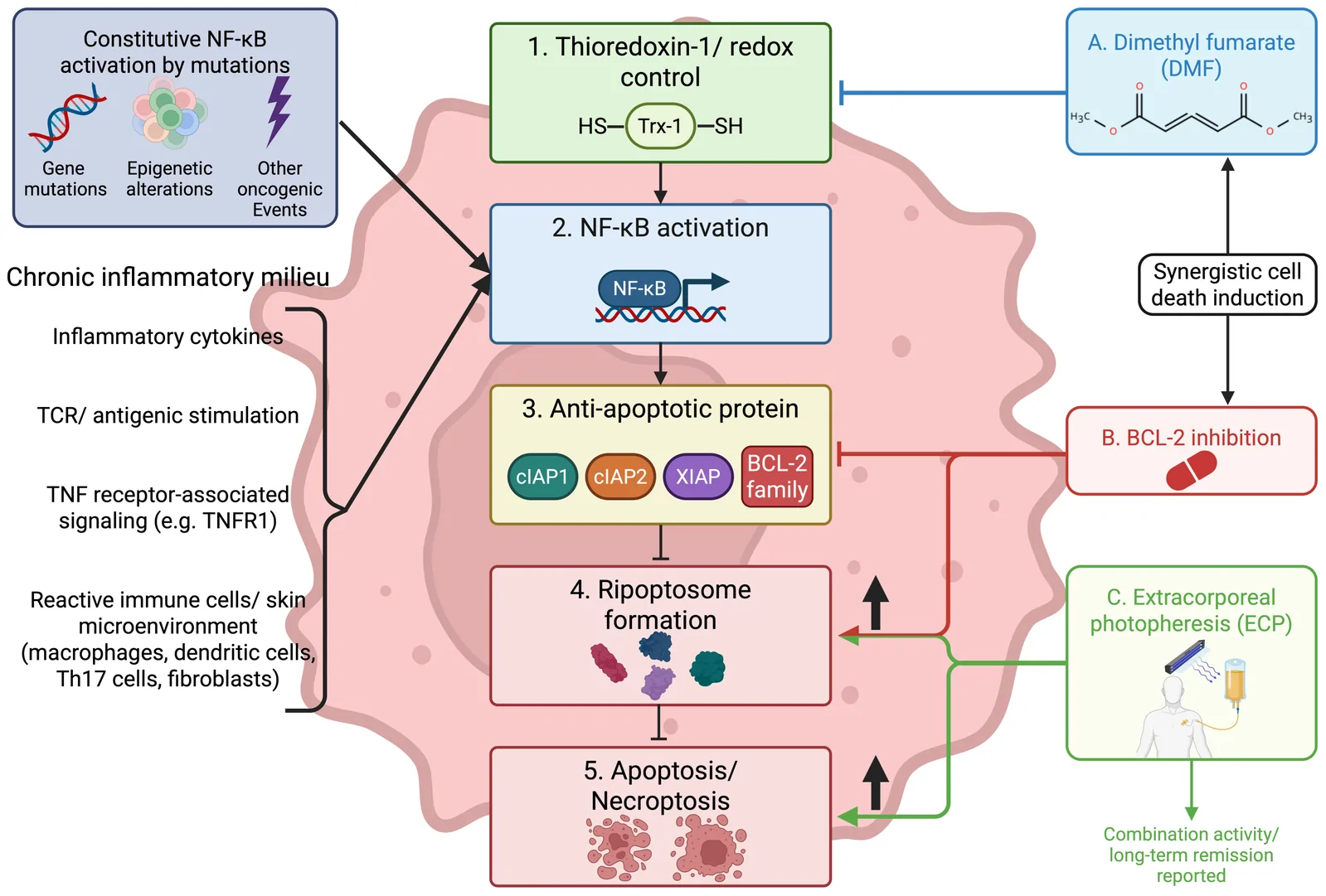
NF-κB, redox control and apoptosis resistance in CTCL. Chronic inflammatory and microenvironmental signals, together with constitutive NF-κB activation driven by genetic or pathway alterations, promote survival signaling in malignant CTCL cells. NF-κB activity supports the expression of anti-apoptotic proteins, including cIAP1, cIAP2, XIAP and BCL-2 family members, which increases resistance to apoptosis and interferes with Ripoptosome formation. Thioredoxin-1 contributes to redox-dependent regulation of NF-κB signaling. Dimethyl fumarate (DMF) is shown as an investigational example of thioredoxin-1 targeting that may reduce NF-κB-dependent survival signaling and restore apoptosis sensitivity. BCL-2 inhibition is likewise investigational in CTCL, whereas extracorporeal photopheresis (ECP) is an established immunomodulatory treatment in selected clinical settings. Created in BioRender. Pollinger, K. (2026) https://BioRender.com/1y6i2yw.

Taken together, targeting NF-κB-driven survival signaling in CTCL is not limited to blocking one transcription factor. It includes interference with upstream inflammatory signals, anti-apoptotic downstream programs, IAP-mediated blockade of Ripoptosome formation, mitochondrial apoptosis control and redox-dependent regulation through thioredoxin-1. These mechanisms help to explain why NF-κB-targeted and related combination strategies are biologically plausible in CTCL and why NF-κB remains an important therapeutic entry point despite the difficulty of directly inhibiting this pathway (Figure 2).

### 5.5. Interface with JAK/STAT Signaling and Immune Dysregulation

Although NF-κB is central, it does not act alone. JAK/STAT signaling is frequently deregulated in CTCL and interacts with inflammatory and apoptotic pathways. Activating alterations in JAK1, JAK3, STAT3, STAT5B and related signaling components have been described in genomic studies, and functional studies indicate that STAT3 and STAT5 can promote survival, cytokine production and immune dysregulation [9,90,91]. Deregulated JAK3/STAT3/STAT5 signaling has also been linked to repression of the tumor suppressor microRNA miR-22, providing an example of how inflammatory signaling can influence gene regulation and malignant T cell behavior [92].

The interaction between NF-κB and JAK/STAT signaling is clinically relevant because both pathways can be activated by cytokines and tissue-derived signals. Inflammatory cytokines may therefore maintain malignant T cell survival through several parallel routes. This helps explain why the inhibition of one pathway may not be sufficient in advanced disease. Therapeutic approaches that target JAK/STAT signaling have shown preclinical activity in CTCL models, and combined or sequential strategies may become relevant in selected patients [90,93]. However, patient selection remains a major challenge, because pathway activation can be driven by mutations, cytokines or microenvironmental signals.

### 5.6. Therapeutic Implications

The close connection between inflammation, NF-κB activation, JAK/STAT signaling and apoptosis resistance has practical therapeutic consequences. Inflammatory signaling should not be viewed only as a background feature of CTCL lesions. It can influence malignant T cell survival, skin inflammation, barrier dysfunction and treatment response. This is supported by studies showing that cytokine-driven signaling contributes to malignant inflammation and tissue dysfunction in CTCL [51,58]. Genomic studies have identified recurrent alterations in genes related to T cell receptor and costimulatory signaling, JAK/STAT signaling, NF-κB pathway regulation and apoptosis control, including alterations affecting PLCG1, CARD11, CD28, STAT3, STAT5B, JAK family members and regulators of cell death [9,10,20]. Thus, inflammatory and survival pathways may be activated by both tumor-intrinsic lesions and signals from the microenvironment.

These considerations have direct implications for patient stratification. Stage, skin tumor burden and blood involvement remain essential for treatment selection, but they do not fully capture malignant-cell biology [7,22]. Surface-target expression, compartment involvement, cytokine responsiveness, apoptotic priming and pathway activation may all influence therapeutic response; the clinical role of CD30-, CCR4- and KIR3DL2-directed therapies is discussed in Section 3, Section 4.4 and Section 7.2. In addition, inflammation-associated immune pathways may provide future therapeutic entry points. The OX40 (CD134)–OX40L axis links T cell costimulation with the tumor microenvironment [94]. Chemokines and chemokine receptors, including the CCR4, CCR10 and CCR7 ligand axes as well as C-X-C chemokine receptor 4 (CXCR4)/C-X-C motif chemokine ligand 12 (CXCL12) and C-X-C chemokine receptor 3 (CXCR3)-associated signaling, contribute to skin tropism, inflammatory-cell recruitment and lymph node trafficking [95,96,97,98]. These pathways are not established therapeutic standards, but they broaden the concept of CTCL treatment from direct cytotoxicity toward modulation of the immune and tissue context in which malignant T cells persist.

Apoptosis resistance remains a central therapeutic problem. Defects in activation-induced cell death, CD95 ligand induction and death receptor signaling can protect malignant T cells from physiological elimination [86,87,88]. In addition, NF-κB-dependent survival signaling can increase the apoptotic threshold of CTCL cells. In this setting, combined NF-κB inhibition and Bcl-2 targeting have synergistically induced CTCL cell death in preclinical models [17]. Together, these findings indicate that restoring apoptosis sensitivity may be most effective when NF-κB-dependent survival signaling is targeted together with mitochondrial apoptosis control.

The therapeutic implications are therefore broader than the selection of a single drug. CTCL treatment increasingly requires an understanding of where the malignant cells are located, which surface targets they express, which inflammatory pathways are active and how resistant they are to cell death. This also means that toxicity and clinical management remain important. For example, mogamulizumab can induce dermatologic adverse events that may mimic or complicate the assessment of CTCL skin disease, making dermatologic expertise and careful monitoring essential [99,100,101,102]. Such issues are particularly relevant in CTCL because skin findings are both a manifestation of the disease and a potential site of treatment-related toxicity.

Taken together, inflammatory signaling and apoptosis resistance are not independent phenomena in CTCL. NF-κB and JAK/STAT signaling can be activated by genetic alterations, cytokines and microenvironmental stimuli, while also increasing the threshold for cell death. These mechanisms help explain the persistence of malignant T cells in chronically inflamed skin and the difficulty of achieving durable disease control in advanced CTCL. They also support treatment strategies that combine compartment-directed therapy, immune modulation and restoration of apoptosis sensitivity rather than relying on cytotoxic activity alone.

## 6. Targeting Oncogenic and Epigenetic Signaling in CTCL

### 6.1. RAS/MAPK Signaling as a Targetable Pathway in Selected CTCL Subsets

The RAS/RAF/MEK pathway is not altered in all patients with CTCL, but it represents an important example of a genotype-linked vulnerability. This pathway regulates proliferation, survival and differentiation in many cell types, including lymphoid cells. In CTCL, high-throughput mutation profiling identified KRAS and NRAS mutations in a subset of samples and showed that RAS-mutant CTCL cells can become sensitive to inhibition of the RAS/RAF/MEK signaling cascade [73,103]. Thus, the study linked RAS pathway mutations in CTCL to a testable therapeutic concept. This is different from surface-directed therapies such as brentuximab vedotin, mogamulizumab or lacutamab, where treatment selection is mainly based on antigen expression. For RAS/MAPK-directed approaches, molecular profiling would be required to identify patients with relevant pathway alterations. The clinical challenge is that RAS/MAPK alterations occur only in a subset of CTCL cases and may not be sufficient as single biomarkers of drug response. Pathway activity can also be influenced by upstream receptor signaling, cytokines and the tumor microenvironment. Therefore, RAS/MAPK inhibition is unlikely to become a broadly applicable strategy for all CTCL patients. At present, it remains a preclinical, molecularly selected therapeutic concept without prospective clinical validation in CTCL [73,103].

### 6.2. Histone Deacetylase Inhibition and Broader Epigenetic Chromatin-Directed Therapy

Epigenetic therapy is already part of the systemic treatment landscape in CTCL. This distinguishes CTCL from many other solid or hematologic malignancies in which epigenetic targeting remains largely experimental. Vorinostat and romidepsin have shown clinical activity in relapsed or refractory CTCL and provide clinical evidence that chromatin-regulated transcriptional programs can be therapeutically exploited in malignant T cells [14,31]. In clinical practice, these agents are not curative, but they have established histone deacetylase inhibition as a relevant treatment principle in advanced CTCL.

Histone deacetylase inhibitors affect more than histone acetylation alone. By changing acetylation of histone and non-histone proteins, they can influence transcription, cell-cycle regulation, apoptosis, immune signaling and cellular differentiation. This broad mode of action may explain why histone deacetylase inhibitors can induce responses in biologically heterogeneous CTCL. At the same time, it also explains some of their limitations. Responses are variable, resistance can develop, and adverse effects such as fatigue, gastrointestinal symptoms, cytopenias, infections or cardiac concerns may restrict long-term treatment [14,31,104]. The clinical activity of histone deacetylase inhibitors also has mechanistic implications. It supports the idea that malignant T cells remain dependent on reversible transcriptional states and not only on fixed genetic lesions. This is relevant in CTCL, where genomic studies have identified recurrent alterations in chromatin remodeling, DNA methylation and histone-modifying pathways [9,10,20]. Epigenetic deregulation may therefore influence cytokine responsiveness, apoptotic threshold, immune phenotype and sensitivity to other therapies.

Epigenetic deregulation in CTCL extends beyond altered histone acetylation [105]. Aberrant DNA methylation, changes in histone marks and dysregulated chromatin-remodeling programs can shape transcriptional states that control T cell identity, cytokine signaling, cell-cycle progression, immune escape and resistance to apoptosis [11]. These processes may contribute both to disease progression and to the heterogeneous activity of epigenetic therapies. In Sézary Syndrome, pharmacological targeting of epigenetic modifiers reduced clonogenic growth and tumor-forming capacity and was associated with the downregulation of human telomerase reverse transcriptase (hTERT), linking epigenetic regulation to malignant-cell persistence [106]. Telomere biology may represent an additional component of this survival network, since CTCL cells express hTERT and show disease-associated patterns of TERT promoter methylation [75]. Together, these findings indicate that sensitivity and resistance to epigenetic therapies are likely determined by the broader transcriptional and chromatin state of the malignant T cell rather than by HDAC activity alone.

Newer chromatin-directed approaches aim to improve this principle. Resminostat, an oral histone deacetylase inhibitor, has been investigated as maintenance therapy in patients with advanced-stage MF or Sézary Syndrome who had achieved disease control with prior systemic treatment. The randomized RESMAIN phase 2 trial supports the idea that epigenetic therapy may be useful not only as induction treatment, but also as a maintenance strategy in selected patients [107]. This concept is clinically attractive in CTCL, where many systemic therapies induce partial or temporary responses and long-term disease control remains difficult. Histone deacetylase inhibition may also be useful in combinations. Preclinical data showed synergy between BCL-2 inhibition and histone deacetylase inhibition in leukemic CTCL cells, suggesting that chromatin-directed therapy can influence apoptotic priming [108]. Other studies have combined JAK inhibition with histone deacetylase inhibition, showing antitumor activity of ruxolitinib plus resminostat in CTCL models [109,110]. These findings suggest that histone deacetylase inhibitors may act not only as single agents, but also as sensitizers for apoptosis- or pathway-directed therapies.

Overall, the clinical activity of HDAC inhibitors and emerging evidence for other epigenetic dependencies show that chromatin regulation is therapeutically relevant in CTCL. The main challenge is not whether chromatin-directed therapy can work, but how to select patients, manage toxicity and define combinations that improve durability without adding unacceptable treatment burden.

### 6.3. EZH2 and Epigenetic Control of Cell Death Sensitivity

Beyond histone deacetylase inhibition, more selective epigenetic regulators may influence how malignant T cells respond to therapy. Enhancer of zeste homolog 2 (EZH2) is the catalytic component of the polycomb repressive complex 2 and mediates trimethylation of histone H3 at lysine 27. Through this mechanism, EZH2 contributes to transcriptional repression and can affect differentiation, proliferation and survival programs in lymphoid malignancies. Although most clinical experience with EZH2 inhibition has been generated in B-cell lymphomas, EZH2 biology is also relevant for T cell immunity and T cell malignancies [111,112,113].

In CTCL, the relevance of EZH2 is not primarily established by recurrent EZH2 mutations, but by functional evidence that EZH2 can influence treatment sensitivity. A genome-wide CRISPR-Cas9 screen identified EZH2 as a regulator of dimethyl-fumarate-induced cell death in malignant T cells. The inhibition of EZH2 sensitized malignant T cells to dimethyl fumarate, indicating that epigenetic repression can help maintain resistance to cell death-inducing stress [61]. This finding is important because it extends the therapeutic relevance of epigenetic regulation beyond established HDAC inhibition and links EZH2 to redox- and NF-κB-associated cell death pathways. These findings suggest that EZH2 can influence apoptosis sensitivity in CTCL cells. Rather than defining EZH2 inhibition as a stand-alone strategy, they support the concept that epigenetic modulation may help sensitize malignant T cells to cell death-inducing therapies. This is of major importance since clinical responses to epigenetic therapy in CTCL are often variable and rarely durable.

## 7. From Molecular Vulnerabilities to Rational Combinations

### 7.1. Why Combinations Are Needed in CTCL

**Figure 3:**
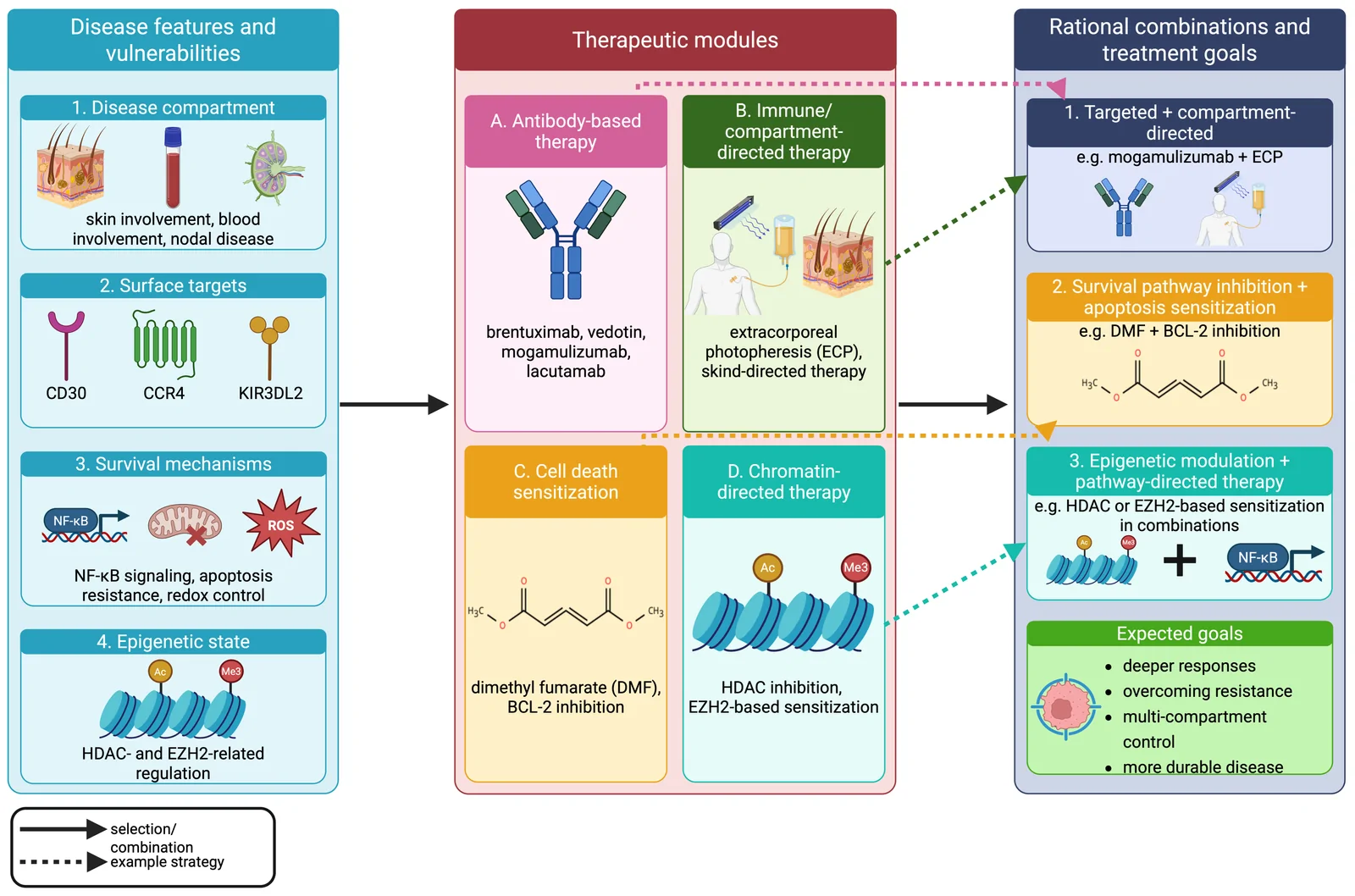
Rational combination strategies in cutaneous T cell lymphoma. Rational combination treatment in cutaneous T cell lymphoma (CTCL) can be guided by clinical disease features and biological vulnerabilities. Relevant variables include disease compartment, such as skin, blood or nodal involvement; surface-target expression, including cluster of differentiation 30 (CD30), CC chemokine receptor 4 (CCR4) and killer cell immunoglobulin-like receptor 3DL2 (KIR3DL2); survival mechanisms such as nuclear factor kappa B (NF-κB) signaling, apoptosis resistance and reactive oxygen species (ROS)-associated redox control; and epigenetic state, including histone deacetylase (HDAC)- and enhancer of zeste homolog 2 (EZH2)-related regulation. These features can be linked to therapeutic modules, including antibody-based therapy, immune- or compartment-directed approaches, cell death sensitization and chromatin-directed treatment. Examples include mogamulizumab combined with extracorporeal photopheresis (ECP), apoptosis sensitization combined with Bcl-2 targeting, and epigenetic modulation combined with pathway-directed therapy. The expected goals are deeper responses, overcoming resistance, improved multi-compartment control and more durable disease control. This figure presents a biological framework for rational combination development and does not imply equivalent clinical evidence or routine clinical applicability of the illustrated approaches. Established treatments should be distinguished from investigational or preclinical pathway-directed combinations, as specified in Table 2 and Table 3. Created in BioRender. Pollinger, K. (2026) https://BioRender.com/1y6i2yw.

Treatment of CTCL cannot rely on one biological principle alone. In early MF, skin-directed therapies can control disease for long periods, but relapses after discontinuation are common and some patients progress despite appropriate local treatment [7,18]. In advanced MF and Sézary Syndrome, malignant T cells may involve several compartments, including skin, blood and lymph nodes. Blood involvement is not only a staging parameter, but also influences prognosis, response assessment and treatment selection [22,62]. This is particularly relevant in Sézary Syndrome, where treatment strategies vary considerably and durable disease control remains difficult. An international observational study of treatment efficacy in the Sézary Syndrome documented substantial heterogeneity of therapeutic approaches and suggested more favorable outcomes with combination therapy than with single-agent treatment [114]. However, the non-randomized design, treatment-selection bias and other potential confounders preclude conclusions regarding comparative superiority. These findings nevertheless support the clinical rationale for further evaluation of biologically and compartment-informed combinations. Mechanistic plausibility alone does not establish synergistic activity or clinical benefit. Whereas synergy has been experimentally demonstrated for selected combinations in preclinical models, several other proposed approaches currently reflect theoretical or clinical complementarity and require prospective clinical validation. At the molecular level, genomic and functional studies show that CTCL cells can rely on several survival mechanisms rather than on a single dominant driver. These include constitutive NF-κB activation and apoptosis resistance, as well as altered redox control, RAS/RAF/MEK signaling and epigenetic regulation (Figure 3) [9,33,59,60].

This helps explain why many systemic treatments produce partial or temporary responses. Conventional chemotherapy can reduce tumor burden, but durable disease control is limited in MF and Sézary Syndrome [16]. Targeted therapies have improved treatment options, but they also illustrate that single agents usually address only part of CTCL biology. Brentuximab vedotin targets CD30-positive disease, mogamulizumab targets CCR4-positive and blood-involved disease, histone deacetylase inhibitors target chromatin-regulated transcriptional programs, and extracorporeal photopheresis is mainly used as an immunomodulatory approach in erythrodermic or blood-involved disease [12,13,26,31]. These treatments are clinically useful, but their different mechanisms also underline that CTCL is rarely controlled by targeting one layer of the disease alone. A clear clinical or biological rationale is essential when considering combinations, but does not replace prospective validation. One treatment may reduce tumor burden in a specific compartment, whereas another may restore apoptosis sensitivity, modify immune function or interfere with inflammatory survival signaling. This differs from the empirical addition of therapies. Biologically plausible examples in CTCL include pairing immune- or compartment-directed treatment with apoptosis sensitization, combining NF-κB inhibition with BCL-2 targeting, or combining extracorporeal photopheresis with agents intended to increase CTCL cell death sensitivity [17,32].

### 7.2. Combining Compartment-Directed and Immune-Based Therapies

One rationale for combination therapy in CTCL is that the disease is distributed across different anatomical and immunological compartments. In early MF, malignant cells are mainly located in the skin, where local inflammation, keratinocyte-derived signals and reactive immune cells contribute to disease persistence. In advanced MF and Sézary Syndrome, malignant T cells may also circulate in the blood and involve lymph nodes. These compartments differ in accessibility, immune context and therapeutic sensitivity. Therefore, a treatment that is effective in one compartment may not fully control disease in another.

This is clinically relevant for antibody-based therapies. Brentuximab vedotin targets CD30-positive CTCL and has shown superior activity compared with physician’s choice in previously treated CD30-positive disease [12,27]. Mogamulizumab targets CCR4 and is particularly relevant in patients with Sézary Syndrome or blood involvement, where circulating malignant T cells are directly accessible to antibody-mediated depletion [13]. More recent analyses of mogamulizumab-treated patients further support that durable responses can occur in selected patients, with clinically meaningful effects on involved disease compartments, especially blood and skin [115]. These findings support the idea that antibody-based therapies should not be viewed only as systemic cytoreductive treatments, but also as compartment-directed strategies.

Extracorporeal photopheresis represents a different but complementary principle. It is mainly used in erythrodermic CTCL and Sézary Syndrome, particularly when blood involvement and immune dysregulation are central features of the disease [26,68]. Unlike antibody-based depletion, extracorporeal photopheresis is not primarily directed against one surface antigen. Its therapeutic effect is thought to involve immune modulation, including effects on antigen-presenting cells, apoptotic cell handling and antitumor immune responses. This makes extracorporeal photopheresis attractive for combinations with agents that increase malignant T cell death or modify inflammatory survival signaling.

Emerging surface targets may further expand this compartment-oriented approach. KIR3DL2 is expressed in Sézary Syndrome and in subsets of MF, and the anti-KIR3DL2 antibody lacutamab showed clinical activity in relapsed or refractory CTCL [15]. Similar to other antibody-based strategies, KIR3DL2-directed treatment depends on the presence of the target antigen on malignant cells. KIR3DL2 has been described as a marker of malignant T cells in Sézary Syndrome and transformed MF, and lacutamab has shown clinical activity in relapsed or refractory CTCL [15,116]. Therefore, KIR3DL2 expression should be considered together with disease subtype, disease compartment and previous therapies when KIR3DL2-directed treatment is evaluated.

Beyond established surface targets, immune-regulatory and trafficking pathways may help to refine future combinations. The OX40–OX40L axis has been discussed as a pathogenic, prognostic and potentially therapeutic pathway in CTCL, linking T cell costimulation with the tumor microenvironment [94]. Chemokines and chemokine receptors contribute to the skin tropism of malignant T cells and to the recruitment of reactive immune cells into CTCL lesions. They may therefore shape the inflammatory microenvironment in which malignant T cells persist [95]. These pathways are not yet equivalent to established therapeutic standards, but they may help to identify patients in whom antibody-based therapy, immunomodulation or microenvironment-directed approaches could be combined more rationally.

Taken together, compartment-directed and immune-based therapies address a central clinical problem in CTCL: malignant cells may persist in skin, blood and lymph nodes under different biological conditions. Rational combinations should therefore consider where the dominant disease burden is located, which surface targets are expressed, and whether the chosen partner treatment modifies immune function, apoptosis sensitivity or inflammatory survival signaling.

### 7.3. Combining Survival-Pathway Targeting with Apoptosis Sensitization

A second rationale for combination therapy in CTCL is the close connection between survival signaling and apoptosis resistance. Constitutive NF-κB activity has been detected in CTCL cells and has been linked to resistance to apoptosis [59,60]. NF-κB can increase the expression of anti-apoptotic and pro-survival factors and may thereby raise the threshold for therapy-induced cell death. Therefore, inhibition of survival signaling alone may be insufficient if mitochondrial apoptosis remains strongly buffered, while direct apoptosis targeting may be less effective if inflammatory survival pathways remain active. These concepts are supported mainly by preclinical and translational studies. Evidence for DMF-based combinations remains limited and does not establish clinical benefit [17,32,33,34,35]. These findings suggest that CTCL cells may become more vulnerable when inflammatory survival signaling, mitochondrial apoptosis control and immune- or compartment-directed treatment are addressed together. Although further validation is needed, these studies support the idea that apoptosis sensitization may strengthen established immunomodulatory or compartment-directed therapies. Overall, combinations that link survival-pathway inhibition with apoptosis sensitization are biologically plausible in CTCL because they address persistent survival signals and protection against programmed cell death. The therapeutic aim is therefore not only to inhibit proliferation or reduce tumor burden, but to lower the threshold for regulated cell death in malignant T cells.

### 7.4. Using Functional Profiling to Guide Combination Treatment

Molecular profiling can identify genetic alterations and targetable surface markers in CTCL, but it does not always predict treatment response. This is particularly relevant for therapies that affect apoptosis sensitivity, redox control or chromatin-regulated transcriptional states. In these settings, drug sensitivity may depend less on a single mutation and more on the current functional state of the malignant T cell. Genomic studies have shown recurrent alterations in chromatin remodeling, DNA methylation and histone-modifying pathways in CTCL, supporting the relevance of epigenetic regulation for disease biology [9,10,20]. However, these findings alone are unlikely to be sufficient for selecting the best combination therapy.

Genomic and transcriptomic profiling can further refine the characterization of CTCL by identifying molecularly distinct tumor states and signaling programs that may not be evident from histology or clinical stage alone. Single-cell RNA sequencing, particularly when combined with T cell receptor sequencing or genomic analysis, has revealed the substantial intratumoral and inter-lesional heterogeneity of malignant T cells and their microenvironment in CTCL [117,118]. DNA methylation profiling may provide an additional layer of information, since aberrant methylation patterns can distinguish Sézary Syndrome from normal T cell populations and may identify clinically relevant epigenetic disease states [74]. These approaches are not yet part of routine treatment selection, but they provide a basis for biomarker-guided trials and for a more precise biological classification of CTCL.

Clinical experience with histone deacetylase inhibitors illustrates this limitation. Vorinostat and romidepsin have shown activity in relapsed or refractory CTCL, but responses are variable and not easily predicted by routine clinical parameters [14,31]. Newer approaches such as resminostat maintenance therapy further support the clinical relevance of chromatin-directed treatment, but they also raise the question of which patients benefit most and which combinations are most useful [107]. Therefore, epigenetic treatment concepts may require additional functional markers of drug sensitivity rather than relying on mutation analysis alone. Functional screening can help identify such therapeutic dependencies. Rather than focusing solely on mutated genes, these approaches determine which genes or pathways malignant cells require to survive under therapeutic pressure. In CTCL, this has been illustrated by a CRISPR-Cas9 screen identifying EZH2 as a regulator of dimethyl-fumarate-induced cell death [61]. This finding is preclinical and does not establish the clinical activity of EZH2 inhibition in CTCL. Since the EZH2 mechanism has been discussed above, the broader implication is that functional screens may reveal combination partners that would not be obvious from routine genomic profiling. Patient-derived CTCL models and drug-screening platforms have additionally identified potentially effective treatment combinations, including combined PI3Kα/δ and HDAC inhibition [119]. Such functional precision-oncology approaches remain investigational but may complement molecular profiling by directly testing treatment vulnerabilities in living tumor cells. Functional approaches are also relevant for apoptosis-directed combinations. Preclinical studies showed synergy between BCL-2 inhibition and histone deacetylase inhibition in leukemic CTCL cells, supporting the idea that chromatin-directed therapy can alter apoptotic priming [108]. Clinical validation of BCL-2-based combinations in CTCL is currently lacking. Similar considerations apply to combinations targeting NF-κB-dependent survival signaling, redox regulation or mitochondrial apoptosis control. These therapeutic dependencies may reflect a cellular state rather than a fixed genetic lesion.

Combination strategies may also exploit interactions between systemic immune-directed treatment and local radiotherapy. Radiotherapy is highly active in CTCL skin lesions and may complement antibody-based therapies by improving local disease control. Beyond local cytoreduction, radiation can modify antigen release, inflammatory signaling and immune recognition, providing a biological rationale for combinations with immune-directed therapies. Available clinical evidence is limited to retrospective analyses and small clinical series. A retrospective multicenter analysis reported the feasibility and clinical activity of simultaneous or sequential brentuximab vedotin and radiotherapy in CD30-positive CTCL [120]. For mogamulizumab, early clinical experience with low-dose total skin electron beam therapy combined with mogamulizumab in refractory MF and Sézary Syndrome suggests that systemic CCR4-directed treatment may be combined with skin-directed radiation to address both blood and skin disease compartments [121]. These approaches are still being defined clinically, but they support the broader principle that rational combinations in CTCL may combine systemic immune- or antibody-based treatment with directed control of the skin compartment.

Future combination strategies in CTCL may therefore need to integrate several levels of information: disease compartment, surface antigen expression, molecular alterations, transcriptional state and functional drug sensitivity. This may include genomic, transcriptomic and methylation profiling, complemented where feasible by single-cell analyses and functional testing of patient-derived tumor cells. The aim is not to add treatments empirically, but to identify combinations in which one component measurably increases sensitivity to another. This would be particularly useful in relapsed or refractory disease, where prior therapy, clonal evolution and changing target expression can make treatment selection difficult (Table 3).

**Table 3:** Mechanism-based therapeutic targets and rational combination concepts in cutaneous T cell lymphoma.

Vulnerability/Biological Layer	Clinical or Biological Context	Therapeutic Implication and Level/Type of Evidence	Example Strategy	Key References
Surface antigen expression	CD30, CCR4 or KIR3DL2 expression; target expression may vary between lesions, patients and disease compartments	Enables antibody-based treatment selection. Evidence: randomized phase III trials support brentuximab vedotin and mogamulizumab; lacutamab is supported by early-phase clinical evidence.	Brentuximab vedotin for CD30-positive disease; mogamulizumab for CCR4-positive or blood-involved disease; lacutamab for KIR3DL2-positive disease	[12,13,15,27]
Disease compartment	Skin-limited disease, erythrodermic CTCL, blood involvement, nodal disease or Sézary Syndrome	Treatment should match the dominant disease compartment and may require combined skin-, blood- or immune-directed approaches. Evidence: guideline recommendations and observational clinical evidence.	Skin-directed therapy, ECP, antibody-based therapy or combined compartment-directed approaches	[5,7,26,65,66]
Radiation-based combination strategies	Extensive symptomatic skin disease or focal refractory lesions requiring local control in addition to systemic treatment	Radiotherapy can provide effective local debulking and may be combined or sequenced with systemic therapy. Evidence: radiotherapy itself is guideline-supported; combination evidence is limited to retrospective analyses and small clinical series, without randomized evidence.	Local radiotherapy or low-dose TSEBT combined or sequenced with brentuximab vedotin or mogamulizumab	[7,19,120,121]
NF-κB-dependent survival signaling	Constitutive inflammatory signaling and apoptosis resistance in malignant T cells	Survival-pathway inhibition may lower the apoptotic threshold. Evidence: preclinical studies and a single-arm phase II study support DMF monotherapy; evidence for DMF-based combinations remains translational and includes a four-patient clinical series for DMF plus ECP.	DMF-based NF-κB inhibition; combination with apoptosis-sensitizing strategies or ECP	[32,33,34,35,59,72]
**Mitochondrial apoptosis control**	Increased anti-apoptotic buffering through BCL-2 family proteins	Direct apoptosis sensitization may enhance treatment-induced cell death. Evidence: preclinical and ex vivo patient-derived data; no prospective clinical trial of BCL-2-based combinations in CTCL.	BCL-2 inhibition combined with NF-κB inhibition or epigenetic modulation	[17,86,87,88]
**Epigenetic regulation**	HDAC-dependent transcriptional programs; EZH2-related cell death resistance	Chromatin-directed therapy may modify apoptotic priming and drug sensitivity. Evidence: HDAC inhibitors are supported by clinical phase II studies, whereas EZH2-based sensitization strategies remain preclinical.	HDAC inhibition; EZH2-based sensitization strategies; chromatin-directed combinations	[14,31,61,107,108]
**RAS/MAPK signaling**	KRAS/NRAS mutations or pathway activation in selected CTCL subsets	Requires molecular selection rather than broad application. Evidence: genomic association and preclinical data; prospective clinical evidence for RAS/MAPK-directed treatment in CTCL is lacking.	RAS/MAPK pathway inhibition in molecularly selected patients	[73,103]
**Functional drug sensitivity**	Vulnerability not predicted by one mutation alone; treatment response may depend on cellular state	Functional profiling may guide rational combinations. Evidence: preclinical and early translational; no functional assay has yet been validated for routine treatment selection in CTCL.	CRISPR-Cas9 screens, ex vivo drug testing, apoptosis priming assays	[10,61,108]

Abbreviations: BCL-2, B-cell lymphoma 2; CCR4, CC chemokine receptor 4; CD30, cluster of differentiation 30; CRISPR-Cas9, clustered regularly interspaced short palindromic repeats-associated protein 9; CTCL, cutaneous T cell lymphoma; DMF, dimethyl fumarate; ECP, extracorporeal photopheresis; EZH2, enhancer of zeste homolog 2; HDAC, histone deacetylase; KIR3DL2, killer cell immunoglobulin-like receptor 3DL2; MF, mycosis fungoides; NF-κB, nuclear factor kappa B; RAS/MAPK, rat sarcoma/mitogen-activated protein kinase; TSEBT, total skin electron beam therapy.

### 7.5. Practical Limitations and Future Directions

Although rational combinations are biologically attractive in CTCL, their clinical implementation remains challenging. Many patients with advanced MF or Sézary Syndrome are older, have received several prior therapies and may have impaired skin barrier function, infections, pruritus, fatigue or systemic immune dysfunction. Adding treatments can therefore increase toxicity, treatment burden and monitoring requirements. This is particularly relevant for combinations that include histone deacetylase inhibitors, antibody-based therapies, extracorporeal photopheresis, retinoids or immune-modulating agents, because each component has its own adverse-effect profile and practical limitations [7,18].

A second limitation is the lack of robust biomarkers for many biologically plausible combinations. Surface markers such as CD30, CCR4 and KIR3DL2 can guide antibody-based treatment, but even these markers may vary between patients, lesions and disease compartments. Other relevant features, such as constitutive NF-κB activation, redox state, mitochondrial apoptotic priming, cytokine responsiveness or epigenetic drug sensitivity, are not yet routinely measured in clinical practice. As a result, many treatment decisions still rely on clinical stage, disease compartment, prior therapy, toxicity profile and physician experience rather than on a fully individualized biological assessment [10,22].

Treatment sequencing is another unresolved issue. Brentuximab vedotin may be preferred in CD30-positive disease, mogamulizumab is particularly relevant in blood-involved disease, extracorporeal photopheresis is often used in erythrodermic CTCL and Sézary Syndrome, and histone deacetylase inhibitors provide an established systemic option in relapsed or refractory disease. However, the optimal order of these therapies is not defined for many clinical scenarios, and most patients receive multiple consecutive treatments with often limited duration of response [7]. Therefore, combinations and sequences should be evaluated not only for response rate, but also for durability, safety, quality of life and feasibility in long-term disease management.

Response assessment can be difficult since skin lesions may improve slowly, relapse can occur in one compartment while another remains controlled, and treatment-related skin reactions may mimic disease activity, particularly with mogamulizumab, where dermatologic adverse events can complicate clinical assessment and require careful distinction from CTCL progression [99]. Such issues underline the need for multidisciplinary evaluation, standardized response criteria and repeated assessment of skin, blood and nodal disease during treatment.

Future studies should therefore move beyond testing combinations empirically. The most useful trials will be those that include a clear biological rationale, defined patient-selection criteria and translational endpoints. These may include repeated assessment of surface markers, molecular profiling in relapsed disease, analysis of blood and skin compartments, and functional assays that test drug sensitivity or apoptosis priming. Such approaches could help identify combinations in which one component genuinely increases the activity of another, rather than simply adding toxicity.

**Figure 4:**
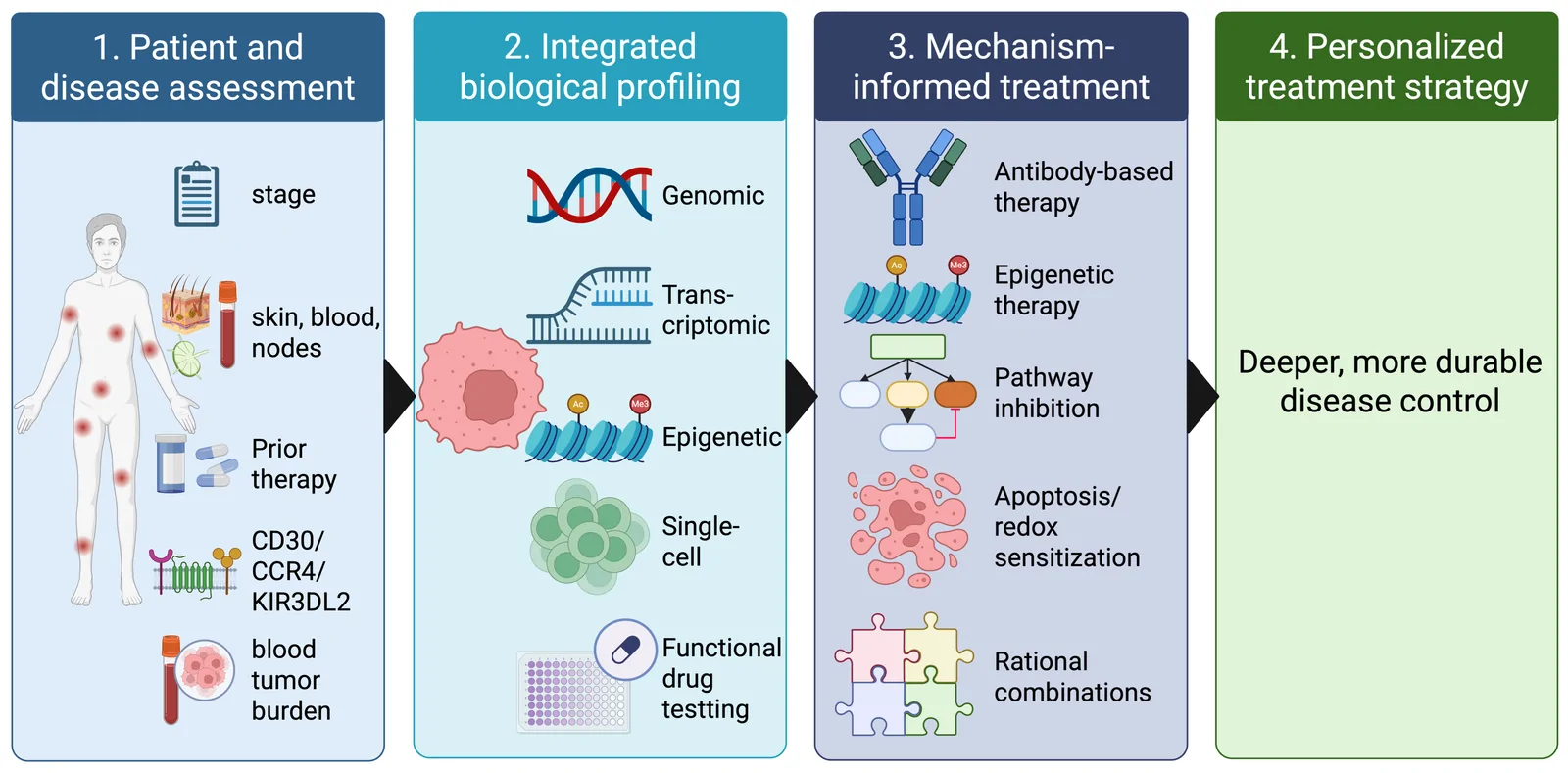
Toward precision medicine in cutaneous T cell lymphoma. Precision medicine in CTCL requires the integration of clinical disease features, including the stage and involvement of skin, blood and lymph nodes, with target expression, molecular and epigenetic profiling, and functional assessment of treatment vulnerabilities. This integrated framework may support the selection of antibody-based, immune-directed, chromatin-directed, pathway-targeted or apoptosis-sensitizing therapies and guide rational combination strategies. The aim is to improve patient stratification and achieve deeper and more durable disease control. Created in BioRender. Pollinger, K. (2026) https://BioRender.com/zea40wk.

In the long term, rational combination therapy in CTCL will require integration of clinical stage, disease compartment, target expression, molecular alterations and functional vulnerability. This is particularly important in relapsed or refractory disease, where clonal evolution, prior therapy and changing microenvironmental conditions may alter treatment sensitivity. The aim should be to develop combinations that are biologically justified, clinically feasible and able to improve durability of response without unacceptable treatment burden (Figure 4).

## 8. Conclusions

CTCL remains difficult to treat because malignant T cells are not sustained by a single dominant driver. Instead, disease persistence and therapy resistance are shaped by several interacting mechanisms, including compartment-specific growth, inflammatory signaling, constitutive NF-κB activation, apoptosis resistance, altered redox control, oncogenic pathway activity and epigenetic regulation. This biological complexity helps explain why many therapies achieve disease control but rarely induce durable remission in advanced MF and Sézary Syndrome. Future treatment concepts should therefore move beyond stage-based escalation alone. Clinical stage, skin and blood involvement, surface-target expression and molecular features need to be considered together with functional vulnerabilities such as apoptotic priming, redox sensitivity and epigenetic drug response. A precision-medicine approach will likely require the integration of genomic, transcriptomic and methylation profiling with single-cell analyses where appropriate, as well as functional drug-sensitivity testing. Together, these approaches may help identify biologically distinct malignant-cell states, define treatment-relevant vulnerabilities and account for heterogeneity across skin, blood and nodal disease compartments. Mechanism-informed strategies, including combinations that link immune modulation, survival-pathway inhibition and apoptosis sensitization, may improve treatment durability if they are guided by clear biological rationale and careful patient selection. Thus, CTCL should increasingly be approached as a disease of malignant T cell survival networks. Functional vulnerability profiling and mechanism-informed combination therapy are not established standards for routine CTCL management. Their translation into clinical practice will require prospective trials, repeated biological reassessment and biomarkers that identify patients most likely to benefit from specific targeted or combination therapies.

## Figures and Tables

**Table 1:** Clinical framework for diagnosis and staging of cutaneous T cell lymphoma.

Domain	Key Features	Diagnostic or Staging Tools	Clinical Relevance	Key References
Clinical presentation	Patches, plaques, tumors, erythroderma; often chronic or relapsing course; distinction between indolent MF and aggressive Sézary Syndrome	Full skin examination; lesion documentation; clinical photography; modified Severity-Weighted Assessment Tool	Defines disease phenotype, skin burden and need for skin-directed versus systemic therapy	[3,5]
Histopathology	Epidermotropic atypical T cells, cerebriform nuclei, variable dermal infiltrates; subtle findings in early MF	Representative skin biopsy; repeat biopsies if needed; dermatopathological review	Supports diagnosis and helps distinguish CTCL from inflammatory dermatoses	[5,19]
Immunophenotype	Mature T cell phenotype; often CD3-positive, CD4-predominant; loss of pan-T cell markers such as CD7 or CD26	Immunohistochemistry; multiparameter flow cytometry	Supports diagnosis; helps detect aberrant malignant T cell populations in skin and blood	[4,19]
T cell receptor clonality	Dominant clonal T cell population; identical clones in separate biopsies or skin and blood strengthen diagnostic confidence	PCR or sequencing-based T cell receptor clonality testing	Provides supportive evidence but must be interpreted with clinicopathological findings	[4,5]
Blood involvement	Aberrant circulating malignant T cells; relevant in erythrodermic CTCL and Sézary Syndrome	Flow cytometry; Sézary cell assessment; T cell receptor clonality; blood-class assessment	Defines blood tumor burden, prognosis and eligibility for specific systemic therapies	[5,7]
Lymph node and visceral assessment	Lymphadenopathy, nodal involvement, rare visceral disease in early MF but relevant in advanced CTCL	Physical examination; imaging; lymph node biopsy when indicated	Distinguishes skin-limited from advanced-stage disease and informs prognosis	[5,6]
TNMB staging	Classification of skin, node, metastasis and blood compartments	TNMB staging system; clinical examination; blood assessment; imaging and pathology as indicated	Provides standardized framework for prognosis, treatment selection and trial comparison	[5,18]
Response assessment	Changes in skin disease, nodal disease, blood involvement and global response	Modified Severity-Weighted Assessment Tool; blood response criteria; nodal and visceral assessment	Allows standardized evaluation of treatment efficacy in clinical practice and trials	[5]
Treatment orientation	Early MF usually treated with skin-directed approaches; advanced MF and SS often require systemic or multimodal treatment	Guideline-based stage-adapted treatment algorithms	Links diagnosis and staging directly to therapeutic decision-making	[7,8]

Abbreviations: CTCL, cutaneous T cell lymphoma; MF, mycosis fungoides; SS, Sézary Syndrome; TCR, T cell receptor; PCR, polymerase chain reaction; TNMB, tumor–node–metastasis–blood; mSWAT, modified Severity-Weighted Assessment Tool; CD, cluster of differentiation.

**Table 2:** Overview of established and emerging therapeutic approaches for cutaneous T-cell lymphoma (CTCL).

	Examples	Preferred Clinical Context	Key Advantages	Main Limitations	References
**Skin-directed therapies**	Topical corticosteroids; phototherapy; topical chlormethine; local radiotherapy; total skin electron beam therapy	Status: guideline-supported standard treatment. Early-stage MF; skin-limited or predominantly cutaneous disease	Evidence: guideline consensus; randomized trial data for topical chlormethine; prospective and retrospective series for TSEBT. Local disease control; limited systemic toxicity; suitable for repeated or maintenance treatment	Incomplete responses; frequent relapse after discontinuation; treatment burden; limited efficacy in advanced systemic disease	[7,19,24]
**Retinoids and rexinoids**	Bexarotene, acitretin	Status: bexarotene is guideline-supported; acitretin is generally off-label. Refractory or advanced-stage CTCL; systemic therapy option, often in combination or sequencing strategies	Evidence: open-label phase II-III trial data for bexarotene. Oral administration; clinical activity in advanced CTCL; improvement in pruritus and CTCL-specific quality of life	Hypertriglyceridemia, hypercholesterolemia, central hypothyroidism; requires monitoring and supportive treatment	[25]
**Immunomodulatory therapies**	Interferon-based therapy; extracorporeal photopheresis	Status: guideline-supported in selected disease settings. Erythrodermic CTCL; Sézary Syndrome; blood involvement; combination strategies	Evidence: guideline consensus, prospective observational studies and retrospective series; limited randomized evidence. Useful in chronic disease control; can be combined with skin-directed and systemic therapies; particularly relevant in Sézary Syndrome	Slow onset; variable response; logistical burden for ECP	[7,26]
**Conventional systemic therapy**	Low-dose methotrexate; gemcitabine; pegylated liposomal doxorubicin; pralatrexate; selected multi-agent chemotherapy	Status: selected regimens are guideline-supported, but several agents are off-label in CTCL. Selected relapsed/refractory cases; rapidly progressive tumor-stage disease; bridging strategies	Evidence: mainly non-randomized phase II studies and retrospective analyses. Cytoreduction can be achieved in selected patients	Responses often short-lived; relevant toxicity; not preferred for durable disease control	[7,16]
**Antibody-based and targeted surface therapies**	Brentuximab vedotin; mogamulizumab; alemtuzumab	Status: regulatory-approved and guideline-supported in relevant disease settings; availability varies by region. CD30-positive CTCL; CCR4-positive or blood-involved MF/Sézary Syndrome	Evidence: randomized phase III trials for brentuximab vedotin and mogamulizumab. Biomarker-linked therapy with improved outcomes in randomized phase III trials	Neuropathy with brentuximab vedotin; infusion reactions, rash and immune-mediated events with mogamulizumab; target heterogeneity	[12,13,27]
**CD52-directed lymphocyte-depleting therapy**	Alemtuzumab	Status: off-label, selected use; not a routine standard option. Relapsed/refractory erythrodermic CTCL or SS with relevant blood involvement	Evidence: small retrospective series and single-center clinical experience. Potential activity in a clinically difficult, heavily pretreated setting	Profound and prolonged immunosuppression; opportunistic infections and viral reactivation; requires prophylaxis and close monitoring	[28,29]
**Immune checkpoint inhibition**	Pembrolizumab	Status: investigational or off-label; no CTCL-specific regulatory approval. Relapsed/refractory MF or SS after prior systemic therapy	Evidence: single-arm multicenter phase II study. Immune-mediated antitumor activity; clinical activity in a multicenter phase II study	Immune-related adverse events and potential disease flares; no established predictive biomarker or standard treatment position in CTCL	[30]
**Epigenetic therapies**	Vorinostat; romidepsin	Status: regulatory approval and guideline support vary by region.	Evidence: pivotal single-arm phase II studies; regulatory approval and guideline support vary by region. Clinical proof of principle that chromatin regulation can be therapeutically exploited	Variable responses; fatigue, gastrointestinal symptoms, cytopenias, infection risk and cardiac concerns may limit use	[14,31]
**Mechanism-informed anti-survival strategies**	Dimethyl fumarate; dimethyl fumarate plus extracorporeal photopheresis	Status: investigational; not guideline-supported. Relapsed/refractory CTCL; investigational or translational treatment setting	Evidence: single-arm phase II study for DMF monotherapy; preclinical and translational evidence, including a four-patient clinical series, for DMF plus ECP. Targets inflammatory and redox-regulated survival pathways; clinical activity reported in phase 2 setting; combination rationale with ECP	Not yet standard guideline therapy; requires further validation and patient stratification	[32,33,34,35]
**Emerging targets and investigational approaches**	Lacutamab/KIR3DL2; pathway inhibitors; apoptosis-targeting agents; redox-modulating strategies	Status: investigational. Advanced, relapsed or refractory CTCL; molecularly or immunophenotypically selected subgroups	Evidence: early-phase trials for lacutamab; predominantly preclinical or early clinical evidence for other approaches. Expands treatment beyond classical staging; may enable more precise or combination-based therapy	Many approaches remain investigational; biomarkers and sequencing are not fully established	[9,10,15]
**Allogeneic hematopoietic stem-cell transplantation**	Allogeneic HSCT	Status: guideline-supported for selected fit patients with advanced-stage or refractory CTCL	Evidence: retrospective multicenter and registry studies; guideline consensus in selected patients. Potentially curative; graft-versus-lymphoma effect	Treatment-related morbidity and mortality; timing and patient selection are critical	[36]
**CAR-T cell therapy**	CAR T cells	Status: experimental; clinical trials only	Evidence: preclinical and early clinical development; no established clinical efficacy in CTCL. Potential future option for selected refractory disease	No established role or curative potential in CTCL; limited clinical evidence; safety and target selection remain unresolved	[37]

Abbreviations: CAR T, chimeric antigen receptor T cell therapy; CCR4, CC chemokine receptor 4; CD30, cluster of differentiation 30; CTCL, cutaneous T cell lymphoma; DMF, dimethyl fumarate; ECP, extracorporeal photopheresis; HSCT, hematopoietic stem-cell transplantation; MF, mycosis fungoides; TSEBT, total skin electron beam therapy.

## Data Availability

No new data were created or analyzed in this study. Data sharing is not applicable to this article.

## Abbreviations

The following abbreviations are used in this manuscript:

**ARID1A**	AT-rich interaction domain 1A
**ATM**	ataxia telangiectasia mutated
**BCL-2**	B-cell lymphoma 2
**BH3**	Bcl-2 homology 3
**CAR**	chimeric antigen receptor
**CARD11**	caspase recruitment domain family member 11
**CCR4**	CC chemokine receptor 4
**CCR7**	CC chemokine receptor 7
**CCR10**	CC chemokine receptor 10
**CDKN2A**	cyclin-dependent kinase inhibitor 2A
**CLIPI**	Cutaneous Lymphoma International Prognostic Index
**CRISPR-Cas9**	clustered regularly interspaced short palindromic repeats-associated protein 9
**CTCL**	cutaneous T cell lymphoma
**CXCL12**	C-X-C motif chemokine ligand 12
**CXCR3**	C-X-C chemokine receptor 3
**CXCR4**	C-X-C chemokine receptor 4
**DMF**	dimethyl fumarate
**DNMT3A**	DNA methyltransferase 3 alpha
**ECP**	extracorporeal photopheresis
**EORTC**	European Organisation for Research and Treatment of Cancer
**ESMO**	European Society for Medical Oncology
**EZH2**	enhancer of zeste homolog 2
**HDAC**	histone deacetylase
**hTERT**	human telomerase reverse transcriptase
**HSCT**	hematopoietic stem-cell transplantation
**IAP**	inhibitor of apoptosis protein
**ICC**	International Consensus Classification
**IL**	interleukin
**ISCL**	International Society for Cutaneous Lymphomas
**JAK**	Janus kinase
**KIR3DL2**	killer cell immunoglobulin-like receptor 3DL2
**MAPK**	mitogen-activated protein kinase
**MEK**	mitogen-activated protein kinase kinase
**MF**	mycosis fungoides
**mSWAT**	modified Severity-Weighted Assessment Tool
**NB-UVB**	narrow-band ultraviolet B
**NCCN**	National Comprehensive Cancer Network
**NFAT**	nuclear factor of activated T cells
**NF-κB**	nuclear factor kappa B
**NGS**	next-generation sequencing
**OX40**	tumor necrosis factor receptor superfamily member 4
**OX40L**	OX40 ligand
**PD-1**	programmed cell death protein 1
**PD-L1**	programmed death-ligand 1
**PI3K**	phosphoinositide 3-kinase
**PLCG1**	phospholipase C gamma 1
**RAF**	rapidly accelerated fibrosarcoma
**RAS**	rat sarcoma viral oncogene homolog
**RELA**	RELA proto-oncogene, NF-κB subunit
**ROS**	reactive oxygen species
**STAT**	signal transducer and activator of transcription
**TCR**	T cell receptor
**TERT**	telomerase reverse transcriptase
**TH2**	T helper 2
**TNF**	tumor necrosis factor
**TNFR**	tumor necrosis factor receptor
**TNMB**	tumor–node–metastasis–blood
**TSEBT**	total skin electron beam therapy
**USCLC**	United States Cutaneous Lymphoma Consortium
**UVA**	ultraviolet A
**WHO-HAEM5**	5th edition of the World Health Organization Classification of Hematolymphoid Tumors
